# Codiscovering graphical structure and functional relationships within data: A Gaussian Process framework for connecting the dots

**DOI:** 10.1073/pnas.2403449121

**Published:** 2024-08-01

**Authors:** Théo Bourdais, Pau Batlle, Xianjin Yang, Ricardo Baptista, Nicolas Rouquette, Houman Owhadi

**Affiliations:** ^a^Computing and Mathematical Sciences, California Institute of Technology, Pasadena, CA 91125; ^b^Jet Propulsion Laboratory, California Institute of Technology, Pasadena, CA 91109

**Keywords:** Gaussian Process, Analysis of variance, hypergraph discovery, raw data analysis, functional relationships

## Abstract

Most problems within and beyond the scientific domain can be framed into one of the following three levels of complexity of function approximation. Type 1: Approximate an unknown function given input/output data. Type 2: Consider a collection of variables and functions, some of which are unknown, indexed by the nodes and hyperedges of a hypergraph (a generalized graph where edges can connect more than two vertices). Given partial observations of the variables of the hypergraph (satisfying the functional dependencies imposed by its structure), approximate all the unobserved variables and unknown functions. Type 3: Expanding on Type 2, if the hypergraph structure itself is unknown, use partial observations of the variables of the hypergraph to discover its structure and approximate its unknown functions. These hypergraphs offer a natural platform for organizing, communicating, and processing computational knowledge. While most scientific problems can be framed as the data-driven discovery of unknown functions in a computational hypergraph whose structure is known (Type 2), many require the data-driven discovery of the structure (connectivity) of the hypergraph itself (Type 3). We introduce an interpretable Gaussian Process (GP) framework for such (Type 3) problems that does not require randomization of the data, access to or control over its sampling, or sparsity of the unknown functions in a known or learned basis. Its polynomial complexity, which contrasts sharply with the super-exponential complexity of causal inference methods, is enabled by the nonlinear ANOVA capabilities of GPs used as a sensing mechanism.

## Significance

Many complex data analysis problems within and beyond the scientific domain involve discovering graphical structures and functional relationships within data. Nonlinear variance decomposition with Gaussian Processes simplifies and automates this process. Other methods, such as artificial neural networks, lack this variance decomposition feature. Information-theoretic and causal inference methods suffer from super-exponential complexity with respect to the number of variables. The proposed technique performs this task in polynomial complexity. This unlocks the potential for applications involving the identification of a network of hidden relationships between variables without a parameterized model at a remarkable scale, scope, and complexity.

**Fig. 1.**
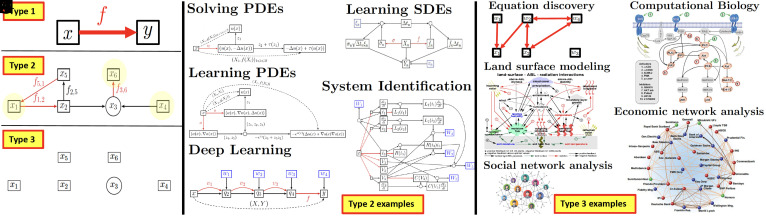
(*A*–*C*) The three levels of complexity of function approximation. *A*: Type 1 (regression). *B*: Type 2 (computational graph completion). *C*: Type 3 (computational hypergraph discovery). (*D*–*H*) Examples of Type 2 problems. *D*: Solving PDEs. *E*: Learning PDEs. *F*: Deep learning. *G*: Learning SDEs. *H*: System identification. (*I*–*M*) Examples of Type 3 problems. *I*: Equation discovery. *J*: Land surface modeling. *K*: Social network analysis. *L*: Computational biology. *M*: Economic network analysis. Image credit: *D*, Reprinted from ref. 12, with permission from Elsevier; *E*-*H*, Reprinted from ref. 7, with permission from Springer Nature; *J*, Reprinted from ref. 26, with permission from Elsevier; *K*, Getty/Ani_Ka; *L*, Reprinted from ref. 29, with permission from the American Association for the Advancement of Science; and *M*, Reprinted from ref. 28, with permission from the American Association for the Advancement of Science.

The three levels of complexity of function approximation. As illustrated in Fig. 1*A*–*C*, Type 1, Type 2, and Type 3 problems can be formulated as completing or discovering hypergraphs where nodes represent variables and edges represent functional dependencies. The graph in Type 1 has only two variables and one unknown function. The graph in Type 2 has multiple variables and (some possibly unknown) functions, and the connectivity of the graph is known. The graph in Type 3 has an unknown connectivity (functional dependencies between variables may be unknown), and this is the focus of this work. Current methods for solving Type 1 and 2 problems include deep learning (DL) methods, which benefit from extensive hardware and software support but have limited guarantees. Despite their prevalence, Type 3 challenges have been largely overlooked due to their inherent complexity. Causal inference methods (1, 2), probabilistic graphs (3, 4), and sparse regression methods (5, 6) offer potential avenues for addressing Type 3 problems. However, it is important to note that their application to these problems necessitates additional assumptions. Causal inference models, for instance, typically assume randomized data and some level of access to the data generation process or its underlying distributions. Sparse regression methods, on the other hand, rely on the assumption that functional dependencies have a sparse representation within a known basis. In this paper, we do not impose these assumptions, and thus, these particular techniques may not be applicable. Furthermore, while the complexity of Bayesian causal inference methods may grow super-exponentially with the number *d* of variables, the complexity of our method is that of *d* parallel computations of polynomial complexities bounded between O(d) (best case) and $O(d^{4})$ (worst case).

## Generalizing Gaussian Process Methods.

Although Gaussian process (GP) methods are sometimes perceived as a well-founded but old technology limited to curve fitting (Type 1 problems), they have recently been generalized, beyond Type 1 problems, to an interpretable framework [Computational Graph Completion or CGC (7)] for solving Type 2 problems (8–13), all while maintaining the simple and transparent theoretical and computational guarantees of kernel/optimal recovery methods (14, 15). This paper introduces a comprehensive GP framework for solving Type 3 problems, which is interpretable and amenable to analysis. This framework leverages the uncertainty quantification (UQ) properties of GP methods, which do not have an immediate natural counterpart in DL methods. It is based on a kernel generalization (16, 17) of variance-based sensitivity analysis guiding the discovery of the structure of the hypergraph. Here, variables are linked via GPs, and those contributing to the highest data variance unveil the hypergraph’s structure. This GP variance decomposition of the data leads to signal-to-noise and a Z-score that can be employed to determine whether a given variable can be approximated as a nonlinear function of a subset of other variables.

## The Scope of Type 1, 2, and 3 Problems.

“Civilization advances by extending the number of important operations we can perform without thinking about them” ( 18). In line with this perspective the scope of Type 1, 2, and 3 problems is immense. Numerical approximation (15, 19–21), Supervised Learning, and Operator Learning (22–25) can all be formulated as Type 1 problems, i.e., as approximating unknown functions given (possibly with noisy and infinite/high-dimensional) inputs/output data. The common GP-based solution to these problems is to replace the underlying unknown function by a GP and compute its MAP estimator given available data. Type 2 problems include (Fig. 1*D*–*H*) solving and learning (possibly stochastic) ordinary or partial differential equations (9, 12), Deep Learning (8), dimension reduction, reduced-ordered modeling, system identification (7), closure modeling, etc. Indeed, all these problems can be formulated as completing a computational graph (7). In this formulation, variables and functions are represented by the nodes and the edges of the graph whose structure corresponds to the functional dependencies between variables. Some of the functions and variables may be unknown, and by completing, we mean approximating the unknown functions (colored in red in Fig. 1) given samples from the observed variables. The common GP-based solution to Type 2 problems is to simply replace unknown functions by GPs and compute their MAP/MLE estimators given available data and constraints imposed by the structure of the graph (7). While most problems in Computational Sciences and Engineering (CSE) and Scientific Machine Learning (SciML) can be framed as Type 1 and Type 2 challenges, many problems in science can only be categorized as Type 3 problems, i.e., discovering the structure/connectivity of the graph itself from data prior to its completion. Indeed the scope of Type 3 problems extends well beyond Type 2 problems and includes equation discovery (Fig. 1*I*); the modeling of land surface interactions in weather prediction (Fig. 1*J* from ref. 26, discovering possibly hidden functional dependencies between state variables for a finite number of snapshots of those variables); social network analysis (Fig. 1*K* from ref. 27, discovering functional dependencies between quantitative markers associated with each individual in situations where the connectivity of the network may be hidden); economic network analysis (Fig. 1*M* from ref. 28, discovering functional dependencies between the economic markers of different agents or companies, which is significant to systemic risk analysis); and computational biology (Fig. 1*L* from ref. 29, identifying pathways and interactions between genes from their expression levels).

## Overview of the Proposed Approach for Type 3 Problems

**Fig. 2.**
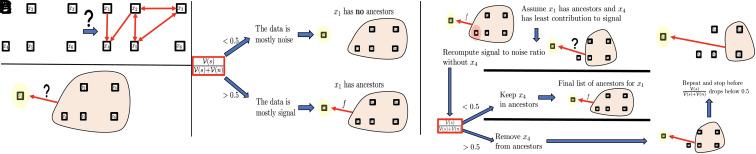
Ancestors identification in Type 3 problem. (*A*) The Type 3 problem under consideration. (*B*) Can *x*_1_ be approximated as a function of $x_{2},\ldots ,x_{6}$? (*C*) Using the signal-to-noise ratio to decide whether *x*_1_ has ancestors. (*D*) Pruning ancestors in decreasing order of contributions to the signal and stopping before the signal-to-noise ratio drops below 0.5.

We first present an algorithmic overview of the proposed GP-based approach for Type 3 problems. For ease of presentation, we consider the simple setting of Fig. 2*A* where we are given *N* samples on the variables $x_{1},\ldots ,x_{6}$. After measurements/collection, these variables are normalized to have zero mean and unit variance. Our objective is to uncover the underlying dependencies between them.

### A Signal-to-Noise Ratio to Decide Whether or Not a Node Has Ancestors.

Our algorithm’s core concept is the identification of ancestors for each node in the graph. Let us explore this idea in the context of a specific node, say *x*_1_, as depicted in Fig. 2*B*. Determining whether *x*_1_ has ancestors is akin to asking if *x*_1_ can be expressed as a function of $x_{2},x_{3},\ldots ,x_{6}$. In other words, can we find a function *f* (living in a prespecified space of functions that could be of controlled regularity) such that:

[1] $$\begin{array}{c} x_{1}\approx f\left(x_{2},\ldots ,x_{6}\right)? \end{array}$$

To answer this question, we regress *f* with a centered GP ξ∼N(0,Γ) whose covariance function Γ is an additive kernel of the form $\Gamma =K_{s}+\gamma \delta \left(x-y\right),$ where *K*_*s*_ is a smoothing kernel, γ>0 is a regularization parameter, and δ(x−y) is the white noise covariance operator. This is equivalent to assuming the GP *ξ* to be the sum of two independent GPs, i.e., $\xi =\xi_{s}+\xi_{n}$ where $\xi_{s}∼N\left(0,K_{s}\right)$ is a smoothing/signal GP and $\xi_{n}∼N(0,\gamma \delta \left(x-y\right))$ is a noise GP. Writing $H_{K_{s}}$ for the reproducing Kernel Hilbert space (RKHS) induced by the kernel *K*_*s*_, this is also equivalent to approximating *f* with a minimizer of

[2] $$\begin{array}{c} \underset{f\in H_{K_{s}}}{\inf}{\left\|f\right\|}_{K_{s}}^{2}+\frac{1}{\gamma }\|f\left(X\right)-Y{\|}_{R^{N}}^{2}, \end{array}$$

where ${\left\|\cdot \right\|}_{R^{N}}^{2}$ is the Euclidean norm on $R^{N}$, *X* is the input data on *f* obtained as an N×5-matrix whose rows *X*_*i*_ are the samples on $x_{2},\ldots ,x_{6}$, *Y* is the output data on *f* obtained as an *N*-vector whose entries are obtained from the samples on *x*_1_, and f(X) is a *N*-vector whose entries are the evaluations $f(X_{i})$. At the minimum

[3] $$\begin{array}{c} V\left(s\right):={\left\|f\right\|}_{K_{s}}^{2} \end{array}$$

quantifies the data variance explained by the signal GP *ξ*_*s*_ and

[4] $$\begin{array}{c} V\left(n\right):=\frac{1}{\gamma }\|f\left(X\right)-Y{\|}_{R^{N}}^{2} \end{array}$$

quantifies the data variance explained by the noise GP *ξ*_*n*_ (17). This allows us to define the signal-to-noise ratio

[5] $$\begin{array}{c} \frac{V(s)}{V(s)+V(n)}\in \left[0,1\right]. \end{array}$$

If $\frac{V(s)}{V(s)+V(n)}<0.5$,^*^ then, as illustrated in Fig. 2*C*, we deduce that *x*_1_ has no ancestors, i.e., *x*_1_ cannot be approximated as function of $x_{2},\ldots ,x_{6}$. Conversely if $\frac{V(s)}{V(s)+V(n)}>0.5$, then, we deduce that *x*_1_ has ancestors, i.e., *x*_1_ can be approximated as function of $x_{2},\ldots ,x_{6}$.

### Selecting the Signal Kernel *K*_*s*_.

This process is repeated by selecting the kernel *K*_*s*_ to be linear ($K_{s}\left(x,x^{\prime }\right)=1+\beta_{1}\sum_{i}x_{i}x_{i}^{\prime }$), quadratic ($K_{s}\left(x,x^{\prime }\right)=1+\beta_{1}\sum_{i}x_{i}x_{i}^{\prime }+\beta_{2}\sum_{i\leq j}x_{i}x_{j}x_{i}^{\prime }x_{j}^{\prime }$) or fully nonlinear to identify *f* as linear, quadratic, or nonlinear. In the case of a nonlinear kernel, we employ:

[6] $$\begin{array}{c} K_{s}\left(x,x^{\prime }\right)=1+\beta_{1}\sum_{i}x_{i}x_{i}^{\prime }+\beta_{2}\sum_{i\leq j}x_{i}x_{j}x_{i}^{\prime }x_{j}^{\prime }+\beta_{3}\prod_{i}\left(1+k\left(x_{i},x_{i}^{\prime }\right)\right), \end{array}$$

where *k* is a universal kernel, such as a Gaussian or a Matérn kernel, with all parameters set to 1, and *β*_*i*_ assigned the default value 0.1. We select *K*_*s*_ as the first kernel that surpasses a signal-to-noise ratio of 0.5. If no kernel reaches this threshold, we conclude that *x*_1_ lacks ancestors.

### Pruning Ancestors Based on Signal-to-Noise Ratio.

**Fig. 3.**
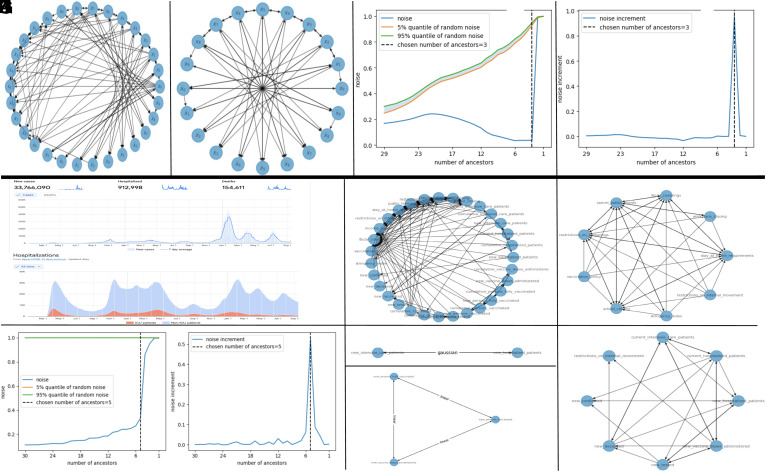
(*A*–*D*) The Fermi–Pasta–Ulam–Tsingou system. (*E*–*K*) The Google COVID-19 open data.

Once we establish that *x*_1_ has ancestors, the next step is to prune its set of ancestors iteratively. We remove nodes with the least contribution to the signal-to-noise ratio and stop before that ratio drops below 0.5 as illustrated in Fig. 2*D*. To describe this, assume that *K*_*s*_ is as in (Eq. **6**). Then *K*_*s*_ is an additive kernel that can be decomposed into two parts:

[7] $$\begin{array}{c} K_{s}=K_{1}+K_{2}, \end{array}$$

where $K_{1}=1+\beta_{1}\sum_{i\neq 1,2}x_{i}x_{i}^{\prime }+\beta_{2}\sum_{i\leq j,i,j\neq 1,2}x_{i}x_{j}x_{i}^{\prime }x_{j}^{\prime }+\beta_{3}\prod_{i\neq 1,2}\left(1+k\left(x_{i},x_{i}^{\prime }\right)\right)$ does not depend on *x*_2_ and $K_{2}=K_{s}-K_{1}$ depends on *x*_2_. This decomposition allows us to express *f* as the sum of two components:

[8] $$\begin{array}{c} f=f_{1}+f_{2}, \end{array}$$

where *f*_1_ does not depend on *x*_2_, *f*_2_ depends on *x*_2_ and $\left(f_{1},f_{2}\right)={\text{argmin}}_{\left(g_{1},g_{2}\right)\in H_{K_{1}}\times H_{K_{2}}\text{s.t.}g_{1}+g_{2}=f}\|g_{1}{\|}_{K_{1}}^{2}+{\left\|g_{2}\right\|}_{K_{2}}^{2}.$ Furthermore, ${\left\|f\right\|}_{K_{s}}^{2}=\|f_{1}{\|}_{K_{1}}^{2}+{\left\|f_{2}\right\|}_{K_{2}}^{2},$ and $\frac{\|f_{2}{\|}_{K_{1}}^{2}}{{\left\|f\right\|}_{K_{s}}^{2}}\in \left[0,1\right]$ quantifies the contribution of *x*_2_ to the signal data variance. Following the procedure illustrated in Fig. 2*D*, if, for example, *x*_4_ is found to have the least contribution to the signal data variance, we recompute the signal-to-noise ratio without *x*_4_ in the set of ancestors for *x*_1_. If that ratio is below 0.5, we do not remove *x*_4_ from the list of ancestors, and $x_{2},x_{3},x_{4},x_{5},x_{6}$ is the final set of ancestors of *x*_1_. If this ratio remains above 0.5, we proceed with the removal. This iterative process continues, and we stop before the signal-to-noise ratio drops below 0.5 to identify the final list of ancestors of *x*_1_. The most efficient version of our proposed algorithm does not use a threshold of 0.5 on the signal-to-noise ratio to prune ancestors, but it rather employs an inflection point in the noise-to-signal ratio $\frac{V(n)}{V(s)+V(n)}\left(q\right)$ as a function of the number *q* of ancestors (Fig. 3*D*). To put it simply, after ordering the ancestors in decreasing contribution to the signal, the final number *q* of ancestors is determined as the maximizer of $\frac{V(n)}{V(s)+V(n)}\left(q+1\right)-\frac{V(n)}{V(s)+V(n)}\left(q\right)$.

### Computational Complexity.

We will now present a detailed analysis of the computational demands of the proposed method as a function of the number of variables, denoted as *d*, and the number of samples, *N*, pertaining to these variables. In the worst case, the proposed approach necessitates, for each of the *d* variables: for i=1,…,d−1, regressing a function mapping d−i variables to the variable of interest and performing a mode decomposition, as exemplified in (Eq. **8**), to identify the variable with the minimal contribution to the signal. Since these two steps have the same cost, it follows that, in the worst case, the total computational complexity of the proposed method is $O(d^{2}N^{3})$ which corresponds to product of the number of double-looping operations, $d^{2}$, and the cost of kernel regression from *N* samples which, without acceleration, is $N^{3}$ (i.e., the cost of inverting a N×N dense kernel matrix). However, if kernel scalability techniques are utilized, such as when the kernel has a rank *k* (for example, k=d if the kernel is linear) or is approximated by a kernel of rank *k* (e.g., via a random feature map), then this worst-case bound can be reduced to $O(d^{2}Nk^{2})$ by reducing the complexity of each regression step from $O(N^{3})$ to $O(Nk^{2})$. Note that the statistical accuracy of the proposed approach requires that N>d if the dependence of the unknown functions on their inputs is not sparse. Moreover, in the absence of kernel scalability techniques, the worst-case memory footprint of the method is $O(N^{2})$ due to the necessity of handling dense kernel matrices. However, once the functional ancestors of each variable are determined, these matrices can be discarded. Consequently, only one such matrix needs to be retained in memory at any given time.

## Results

The following examples and experiments illustrate the proposed approach.

### The Fermi–Pasta–Ulam–Tsingou System.

The Fermi–Pasta–Ulam–Tsingou (FPUT) system (30) is a prototypical chaotic dynamical system. It is composed of *M* masses indexed by j∈{0,…,M−1} with equilibrium position jh with h=1/M. Each mass is tethered to its two adjacent masses by a nonlinear spring, and the displacement of the mass *x*_*j*_ adheres to the equation:

[9] $$\begin{array}{c} {\ddot{x}}_{j}=\frac{c^{2}}{h^{2}}\left(x_{j+1}+x_{j-1}-2x_{j}\right)\left(1+\alpha (x_{j+1}-x_{j-1})\right), \end{array}$$

where $\alpha \left(x\right)=x^{2}$, c=1 and M=10. We use fixed boundary conditions by adding two more masses, with $x_{-1}=x_{M}=0$. We take a total of 1,000 snapshots from multiple trajectories and the observed variables are the positions, velocities, and accelerations of all the underlying masses. In the graph discovery phase, every other node is initially deemed a potential ancestor for a specified node of interest. We then proceed to iteratively remove the node with the least signal contribution. The step resulting in the largest surge in the noise-to-signal ratio is inferred as one eliminating a crucial ancestor, thereby pinpointing the final ancestor set. Fig. 3*C* shows a plot of the noise-to-signal ratio $\frac{V(n)}{V(s)+V(n)}\left(q\right)$ as a function of the number *q* of proposed ancestors for the variable ${\ddot{x}}_{7}$ and with *Z*-test quantiles (in the absence of signal, the noise-to-signal ratio should fall within the shaded area with probability 0.9). Removing a node essential to the equation of interest causes the noise-to-signal ratio to markedly jump from approximately 25% to 99%. Fig. 3*D* shows a plot of the noise-to-signal ratio increments $\frac{V(n)}{V(s)+V(n)}\left(q\right)-\frac{V(n)}{V(s)+V(n)}\left(q-1\right)$ as a function of the number *q* of ancestors for the variable ${\ddot{x}}_{7}$. Note that the increase in the noise-to-signal ratio is significantly higher compared to previous removals when an essential node was removed. Therefore, while solely relying on a fixed threshold to decide when to cease the removals might prove challenging, evaluating the increments in noise-to-signal ratios offers a clear guideline for efficiently and reliably pruning ancestors. The recovered full graph, depicted in Fig. 3*A*, is remarkably accurate despite the nonlinear nature of the model and the fact that our prior only encodes that the nonlinearity is smooth. Therefore, our algorithm does not require a dictionary or extensive knowledge of the structure of the unknown functions. Notably, velocity variables are accurately identified as nonessential and omitted from the ancestors of position and acceleration variables. Fig. 3*B*, which omits velocity variables for clarity, further elucidates the accurate recovery of dependencies. The dependencies are the simplest and clearest possible. They match exactly those of the original equations except for the boundary particles for which we recover valid equivalent equations.

### The Google COVID-19 Open Data.

Consider the COVID-19 data from Google.^†^ We focus on a single country, France, to ensure consistency in the data and avoid considering cross-border variations that are not directly reflected in the data. We select 31 variables that describe the state of the country during the pandemic, spanning over 500 data points, with each data point corresponding to a single day. These variables are categorized as the following datasets: 1) Epidemiology dataset: Includes quantities such as new infections, cumulative deaths, etc. 2) Hospital dataset: Provides information on the number of admitted patients, patients in intensive care, etc. 3) Vaccine dataset: Indicates the number of vaccinated individuals, etc. 4) Policy dataset: Consists of indicators related to government responses, such as school closures or lockdown measures, etc. Some of these variables are illustrated in Fig. 3*E*. The problem is then to analyze this data and identify possible hidden functional relations between these variables. Fig. 3*F* shows the noise-to-signal ratio (and its increments) as function of the number of ancestors of the “cumulative number of hospitalized patients” variable. Even for this real dataset, the proposed approach gives a clear signal for stopping the pruning process. Fig. 3*G* shows the full recovered graph, which is highly clustered. Fig. 3*H* shows the cluster corresponding to the variable “schools closing” revealing that the government either implemented multiple restrictive measures simultaneously or lifted them in unison (except for mask mandates that were on the verge of being identified as noise). The vaccination cluster (Fig. 3*J*) reveals a linear relationship between variables (signaling redundant information) and the hospitalization cluster (Fig. 3*I*) reveals a nonlinear one. Eliminating redundant nodes leads to the sparse graph shown in Fig. 3*K*, which is interpretable and amenable to (both quantitative and qualitative) analysis,

### Chemical Reaction Network.

**Fig. 4.**
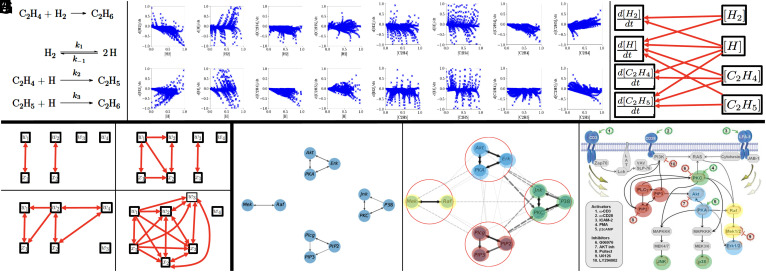
(*A*–*C*) Chemical reaction network. (*D*–*G*) Algebraic equations. (*H*–*J*) Cell signaling network (Image credit: *J*, Reprinted from ref. 29, with permission from the American Association for the Advancement of Science).

In this example, we consider the recovery of a chemical reaction network from concentration snapshots. The reaction network, illustrated in Fig. 4*A*, is that of the hydrogenation of ethylene $\left({\text{C}}_{2}{\text{H}}_{4}\right)$ into ethane $\left({\text{C}}_{2}{\text{H}}_{6}\right)$. The problem is that of recovering the underlying chemical reaction network from snapshots (illustrated in Fig. 4*B*) of concentrations $\left[H_{2}\right]$, [H], $\left[C_{2}H_{4}\right]$, and $\left[C_{2}H_{5}\right]$ and their time derivatives. $\frac{d[H_{2}]}{\mathrm{dt}}$, $\frac{d[H]}{\mathrm{dt}}$, $\frac{d[C_{2}H_{4}]}{\mathrm{dt}}$ and $\frac{d[C_{2}H_{5}]}{\mathrm{dt}}$. The proposed approach leads to a perfect recovery of the computational graph (shown in Fig. 4*C*) and a correct identification of quadratic functional dependencies between variables.

### Algebraic Equations.

Fig. 4*A*–*D* illustrates the application of the proposed approach to the recovery of functional dependencies from data satisfying hidden algebraic equations. In all these examples, we have d=6 or d=7 variables and N=1,000 samples from those variables. For d=6 the variables are $w_{1},w_{2},w_{3},w_{4},x_{1},x_{2}$. For d=7 the variables are $w_{1},w_{2},w_{3},w_{4},x_{1},x_{2},x_{3}$. The samples from the variables *w*_1_ to *w*_4_ are i.i.d. N(0,1) random variables, and the samples from *x*_1_, *x*_2_ (and *x*_3_ for d=7) are functionally dependent on the other variables. In the first example, d=6 and the samples from *x*_1_ and *x*_2_ satisfy the equations $x_{1}=w_{1}$ and $x_{2}=w_{2}$. The algorithm selects the linear kernel, and Fig. 4*A* shows the recovered graph (which is exact). In the second example, d=7 and the samples from $x_{1},x_{2}$, and *x*_3_ satisfy the equations $x_{1}=w_{1}$, $x_{2}=x_{1}^{2}+1+0.1w_{2}$, and $x_{3}=w_{3}$. The algorithm selects the quadratic kernel and Fig. 4*B* shows the recovered graph (which is exact). Even though *x*_2_ can trace back its origin to either *x*_1_ and *w*_2_ or *w*_1_ and *w*_2_, the algorithm recognizes *x*_1_, *w*_1_, and *w*_2_ as its ancestors underscoring the importance of eliminating redundant variables when aiming at deriving the sparsest graph. In the third example, d=6 and the samples from *x*_1_ and *x*_2_ satisfy the equations $x_{1}=w_{1}w_{2}$ and $x_{2}=w_{2}\sin\left(w_{4}\right)$. The algorithm selects the nonlinear kernel and Fig. 4*C* shows the recovered graph (which is exact). In the fourth example, d=7 and the samples from $x_{1},x_{2}$, and *x*_3_ satisfy the equations $x_{1}=w_{1}$, $x_{2}=x_{1}^{3}+1+0.1w_{2}$ and $x_{3}={\left(x_{1}+2\right)}^{3}+0.1w_{3}$. Although these equations appear to be cubic, the algorithm correctly selects the quadratic kernel and makes an exact recovery of the graph shown in Fig. 4*D* revealing hidden quadratic dependencies between variables.

### Cell Signaling Network.

Next, we apply the proposed framework to the example illustrated in Fig. 1*L* from ref. 29 and discover a hierarchy of functional dependencies in biological cellular signaling networks. We use single-cell data consisting of the d=11 phosphoproteins and phospholipids levels in the human immune system T cells that were measured using flow cytometry. This dataset was studied from a probabilistic modeling perspective in previous works. While Sachs et al. (29) learned a directed acyclic graph to encode causal dependencies, Friedman et al. (31) learned an undirected graph of conditional independencies between the *d* molecule levels by assuming the underlying data follows a multivariate Gaussian distribution. The latter analysis encodes acyclic dependencies but does not identify directions. In this work, we aim to identify the functional dependencies without imposing strong distributional assumptions on the data. We simply use N=2,000 samples chosen uniformly at random from the dataset consisting of 11 proteins and 7,446 samples of their expressions. We apply the algorithm in two stages. The first stage of the algorithm uses only linear and quadratic kernels and recovers the graph shown in Fig. 4*H*. It consists of four disconnected clusters where the molecule levels in each cluster are closely related by linear or quadratic dependencies (all connections are linear except for the connection between Akt and PKA, which is quadratic). These edges match a subset of the edges found in the gold standard model identified in ref. 29. With perfect noiseless dependencies, one can define constraints that reduce the total number of variables in the system. Second, we learn the connections between groups of variables within each cluster with nonlinear kernels and obtain the graph shown in Fig. 4*I* in which solid arrows indicate strong intracluster connections identified in the first level, and dashed lines indicate weaker connections between nodes and clusters identified in the second level. The width and grayscale intensities of each edge correspond to its signal-to-noise ratio. We emphasize that while causal graph recovery methods rely on the control of the sampling of the underlying variables (i.e., the simultaneous measurement of multiple phosphorylated protein and phospholipid components in thousands of individual primary human immune system cells, and perturbing these cells with molecular interventions), the reconstruction obtained by our method did not use this information and recovered functional dependencies rather than causal dependencies. Interestingly, the information recovered through our method appears to complement and enhance the findings presented in ref. 29 (e.g., the linear and noiseless dependencies between variables in the JNK cluster is not something that could easily be inferred from the graph produced in ref. 29 shown in Fig. 1*J* where we have colored the clusters for comparison).

#### Comparisons.

**Fig. 5.**
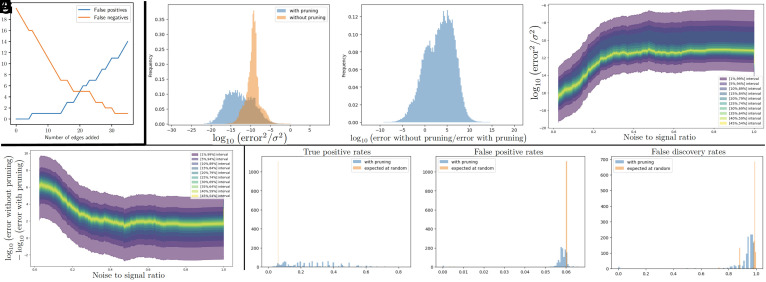
(*A*) Cell signaling network comparisons. (*B*–*H*) The BCR reaction benchmark.

Using the expected graph reported in ref. 29 as the ground truth (acknowledging that it may not be entirely accurate), we compare the edges our approach incrementally added to the true graph. Fig. 5*A* reports the number of additional edges that have been added and are not present in the ground truth (false positives) and edges removed that are present in the ground truth graph (false negatives). The added edges are based on the two-stage procedure described above, where we first add the ten intracluster connections, followed by intercluster connections. Edges are added in decreasing order of signal-to-noise ratio, starting with the strongest. In the reported results, we do not account for the recovery of the direction of ground-truth edges. We note that, up to direction, all intracluster connections, along with the intercluster connections with the strongest signals are found in the ground truth graph, leading to the initial decrease in false negatives with only one false positive edge (the linear connection P38 → Jnk that is not reported in the true graph). With the addition of the remaining (possibly nonspurious) edges, the number of false negatives drops to one, having recovered all edges, except for the one between PKC and Raf, which is identified to be statistically noninformative in our approach.

### A Large-Scale Chemical Reaction Network: The BCR Reaction Benchmark.

Finally, we stress-test the scalability of our approach by applying it to a large-scale chemical reaction network: the BCR reaction benchmark from ref. 32, which encompasses 1,122 species. The dataset comprises 2,400 snapshots of species concentrations and their corresponding time derivatives. We leveraged JAX’s inherent parallelization capabilities (33) to accelerate our computations, allowing for the simultaneous pruning of multiple nodes while abstracting the complexity of parallel execution. While the scaling with respect to the number of data points is straightforward, scaling with the number of variables introduces a trade-off between computational speed and memory footprint. Specifically, the process of identifying the ancestors of various nodes can be expedited by storing a large array for all nodes. Using a DGX workstation equipped with four Nvidia V100 GPUs, each with 32GB of memory, pruning 190 nodes took approximately three days, projecting a total experiment duration of around one month. Nonetheless, we can mitigate this computational burden by optimizing the computation of terms of the form $y^{T}Ky$ for the specific quadratic kernel identified for this example. We include the details of such optimization in *SI Appendix*, section 8E. By implementing this optimization, the duration of the entire experiment was reduced to just 1 h.

In the first experiment, we simulated five trajectories of the associated system of ODEs, recording 1,000 snapshots per trajectory. Out of these 5,000 snapshots, 2,400 were randomly selected as training data, and 2,600 as testing data. Writing TP, TN, FP, and FN for True/False Positives/Negatives and using the metrics True Positive Rate (TPR=TP/(TP+FN)), False Positive Rate (FPR=FP/(FP+TN)), and false discovery rate (FDR=FP/(TP+FP)), we observed a TPR of 39.9%, an FPR of 16.4%, and an FDR of 97.2% (indicating that 97.2% of predicted positives are false). This high FDR can be attributed to the limited exploration of the full variable range-1,122 in total-by the five trajectories. The trajectories explored a subset of the possible space (near a limit cycle attractor), which led to the recovery of functional dependencies that represent both the chemical reactions and the specific subspace visited. Furthermore, with 1,122 variables, the 630,003 coefficients of the underlying quadratic equations are vastly underdetermined with only 2,400 data points. Despite the high FDR in the recovered graph, as illustrated in Fig. 5 *B* and *C*, the CHD pruning process vastly improves the accuracy (by orders of magnitude) of the estimated functions on the 2,600 unseen snapshots by reducing the dimension of the regression problem whenever possible. We denote *y*_*i*_ as an observed data point, $\sigma^{2}$ as the variance of the observed data, ${\hat{y}}_{i}$ for a predicted data point without pruning, and ${\overset{¯}{y}}_{i}$ for a predicted data point postpruning. Fig. 5*B* illustrates the histogram of the log-normalized squared errors before and after pruning, expressed as $\log_{10}({\left|y_{i}-{\hat{y}}_{i}\right|}^{2}/\sigma^{2})$ and $\log_{10}({\left|y_{i}-{\overset{¯}{y}}_{i}\right|}^{2}/\sigma^{2})$. The 99th percentile of the normalized squared error is less than $10^{-2}$ for all species. Fig. 5*C* displays the histogram of the log-normalized squared error improvements due to pruning, calculated as $\log_{10}(|y_{i}-{\hat{y}}_{i}{|}^{2}/{\left|y_{i}-{\overset{¯}{y}}_{i}\right|}^{2})$. Fig. 5 *D* and *E* displays the quantiles of the histograms postpruning, conditioned on the noise-to-signal ratio observed at the final pruning step. These plots reveal a clear trend: a higher noise-to-signal ratio at the time of pruning correlates with increased error and diminished improvements in accuracy.

In a second experiment, we formed the data by randomly sampling concentrations uniformly in [0,1] (independently across species and snapshots) and recorded the resulting time derivatives. While this sampling increased the variability of the 2,400 snapshots, the model remained vastly underdetermined. The noise-to-signal and bootstrapped (Z-test) ratios remained close to 0.5, suggesting insufficient data for statistically significant variable importance assessments. Nonetheless, as depicted in Fig. 5*F*–*H*, significant insights can still be gleaned from the activations, showing notable improvements when comparing the histograms of the values of TPR, FPR, and FDR obtained with pruning based on these ratios and pruning at random. This analysis reveals that even with high dimensionality and scarce data, between 10% and 80% of the true ancestors can still be accurately identified.

## Discussions

### Limitations.

In its present form, the proposed approach is limited by several factors. 1) Without access to the sampling of the data, the direction of some edges may not be identifiable. For instance, the functional relationship x−2y=0 can be represented as both y=2x (x→y) and x=y/2 (y→x). 2) It assumes an additive noise *W* on the functional relationship y=f(x)+W between the variables *x* and *y*. In a fully probabilistic setting, this structure may be nonadditive, i.e., of the form y=f(x,W), which implies discovering a general transition kernel, i.e., a non-Gaussian generative model. Although our method achieves polynomial complexity, in settings where one has access to the distribution of the data, the price to pay, when compared with information-theoretic methods, is a reduction in generality imposed by the stronger assumption made on the data-generating process. Furthermore, the price to pay for the weaker data requirements (i.e., the absence of interventional data) is that our method recovers functional relationships rather than causal ones or conditional dependencies. 3) If the (noisy) functional relationship y=f(x)+W is associated with a nonregular (e.g., discontinuous) function *f* then the kernels discussed above (linear, quadratic, and fully nonlinear) will be misspecified and may lead to false negatives. The kernel selection and hyperparameter tuning problems in misspecified settings require further work. 4) As demonstrated in the BCR reaction application, while the method scales well computationally with an increase in the number of variables, it may still be impacted by the curse of dimensionality. This occurs particularly if the dataset only covers a limited subset of the full range of variable values. Given the results displayed in Fig. 5*B*–*H*, we suspect that this impact could be mitigated by adopting more advanced strategies in place of our current top–down pruning method. Such strategies could involve grouping variables and integrating both top–down and bottom–up iterative approaches.

### Conclusions.

We have developed a comprehensive GP framework for solving Type 3 (hypergraph discovery) problems, which is interpretable and amenable to analysis. The breadth and complexity of Type 3 problems significantly surpass those encountered in Type 2 (hypergraph completion), and the initial numerical examples we present serve as a motivation for the scope of Type 3 problems and the broader applications made possible by this approach. Our proposed algorithm is designed to be fully autonomous, yet it offers the flexibility for manual adjustments to refine the graph’s structure recovery. We emphasize that our proposed approach is not intended to supplant causal inference methods (34); see *SI Appendix*, section 4C for a complete overview. Instead, it aims to incorporate a distinct kind of information into the graph’s structure, namely, the functional dependencies among variables rather than their causal relationships. Additionally, our method eliminates the need for a predetermined ordering of variables, a common requirement in acyclic probabilistic models where determining an optimal order is an NP-hard problem usually tackled using heuristic approaches. Furthermore, our approach can actually be utilized to generate such an ordering by quantifying the strength of the connections it recovers. The Uncertainty Quantification properties of the underlying GPs are integral to the method and could also be employed to quantify uncertainties in the structure of the recovered graph. We also observe that forming clusters from highly interdependent variables helps to obtain a sparser graph. Additionally, the precision of the pruning process is enhanced by avoiding the division of node activation within the cluster among its separate constituents. We employed this strategy in the recovery of the gene expression graph in Fig. 4*I*. Given the polynomial complexity of our method, promising avenues for future work include applications to large datasets in genomics and in systems biology, particularly in the reconstruction and intervention of metabolic pathways. These applications benefit from the ability to handle large-scale datasets efficiently, enabling the analysis of complex biological networks.

## Supplementary Material

Appendix 01 (PDF)

## Data Availability

Data and Python Code data [the code for the algorithm and its application to various examples are available for download (and as an installable python library/package)] have been deposited in Github (https://github.com/TheoBourdais/ComputationalHypergraphDiscovery).

## Footnotes

^*^We will later present a version with a more sophisticated method for pruning, but we keep the 0.5 threshold in this example for simplicity.

^†^The dataset can be accessed at https://health.google.com/covid-19/open-data/raw-data.
