# Supplementary material for: Codiscovering graphical structure and functional relationships within data: A Gaussian Process framework for connecting the dots
Source: Proc Natl Acad Sci U S A. 2024 Aug 1;121(32):e2403449121. doi: 10.1073/pnas.2403449121 (PMC11317615; doi:10.1073/pnas.2403449121)
Supplement: Supplementary file 1 — Appendix 01 (PDF) [file pnas.2403449121.sapp.pdf]

PNAS

Supporting Information for

Co-discovering Graphical Structure and Functional Relationships Within Data: A Gaussian Process Framework for Connecting the Dots

Théo Bourdais, Pau Batlle, Xianjin Yang, Ricardo Baptista, Nicolas Rouquette, Houman Owhadi

Houman Owhadi
E-mail: owhadi@caltech.edu

This PDF file includes:

Supporting text
Figs. S1 to S9
SI References

Supporting Information Text

This supplementary document provides an overview of refinements and generalizations on our proposed approach (Sec. 1) detailed in subsequent sections. It includes a summary of the principal components of our algorithm (Sec. 2). It includes a reminder on Type 2 problems (Sec. 3) and their common GP-based solutions. It discusses the hardness of Type 3 problems, presents an overview of causal inference methods, and a well-posed formulation of Type 3 problems (Sec. 4). Additionally, this document offers an in-depth description of our developed GP-based solution specifically designed for Type 3 problems (Section 5), along with the corresponding algorithmic pseudo-codes (Section 6). It also includes an analysis of the signal-to-noise ratio (SNR) test that is integral to our method (Section 7), and furnishes supplementary details concerning the examples discussed in the main manuscript (Section 8).

1. Additional details on our proposed approach.

The efficacy of our proposed approach is enhanced through a series of refinements (implemented in all our examples), which are summarized below and detailed in sections 5, 6 and 7.

A. Ancestor pruning.. As discussed earlier, rather than using a threshold on the signal-to-noise ratio to prune ancestors, we order the ancestors in decreasing contribution to the signal, the final number q of ancestors is determined as the maximizer of noise to signal ratio increment $\frac{\mathcal{V}(n)}{\mathcal{V}(s)+\mathcal{V}(n)}(q+1) - \frac{\mathcal{V}(n)}{\mathcal{V}(s)+\mathcal{V}(n)}(q)$.

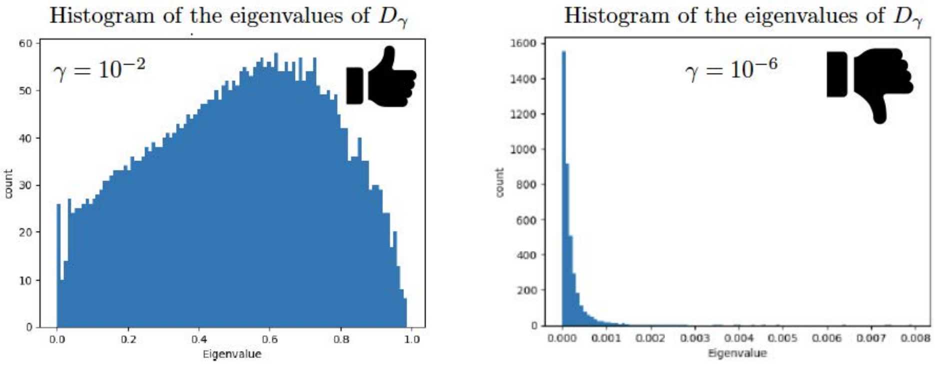

Fig. S1. Histogram of the eigenvalues of D_γ=Eq. (10) for $\gamma = 10^{-2}$ (good choice) and $\gamma = 10^{-6}$ (bad choice).

B. Parameter Selection.. The choice of the parameter γ in Eq. (2)is a critical aspect of our proposed approach. We provide a structured approach for selecting γ based on the characteristics of the kernel matrix K_s. Specifically, when K_s is derived from a finite-dimensional feature map ψ (i.e., when $K_s(x,x') := \psi(x)^T\psi(x')$ where the range of ψ is finite-dimensional) and the data cannot be interpolated exactly with K_s (the dimension of the range of ψ is smaller than the number of data points), we employ the regression residual to determine γ as follows:

$$\gamma = \min_v \left\| v^T\psi(X) - Y \right\|^2_{\mathbb{R}^N} . \qquad [9]$$

Write $K_s(X,X)$ for the $N \times N$ matrix with entries $K_s(X_i, X_j)$. Alternatively, when the data can be interpolated exactly with K_s (e.g., when K_s is a universal kernel), we select γ (see Fig. S1) by maximizing the variance of the eigenvalue histogram of the $N \times N$ matrix

$$D_\gamma := \gamma\big(K_s(X,X) + \gamma I\big)^{-1} , \qquad [10]$$

whose eigenvalues are bounded between 0 and 1 and converge towards 0 as $\gamma \downarrow 0$ and towards 1 as $\gamma \uparrow \infty$. We can also select γ as the median of the eigenvalues of D_γ.

C. Z-test quantiles.. The noise-to-signal ratio $\frac{\mathcal{V}(n)}{\mathcal{V}(s)+\mathcal{V}(n)}$ associated with Eq. (2)admits the representer formula $\frac{Y^T D_\gamma^2 Y}{Y^T D_\gamma Y}$. Therefore if the data is only comprised of noise (if $Y \sim \sigma^2 Z$ where Z is a random vector with i.i.d. $\mathcal{N}(0,1)$ entries), then the distribution of the noise-to-signal ratio follows that of the random variable

$$B := \frac{Z^T D_\gamma^2 Z}{Z^T D_\gamma Z} . \qquad [11]$$

Therefore, the quantiles of B can be used as an interval of confidence on the noise-to-signal ratio if $Y \sim \sigma^2 Z$. Fig. 3.(c) shows these Z-test quantiles (in the absence of signal, the noise-to-signal ratio should fall within the shaded area with probability 0.9).

D. Generalizations on our proposed approach..

D.1. Complexity Reduction with Kernel PCA Variant.. Write K for the kernel associated with the RKHS $\mathcal{H}$ in Problem 1. We use a variant of Kernel PCA (1) to significantly reduces the computational complexity of our proposed method, making it primarily dependent on the number of principal nonlinear components in the kernel matrix $K(X,X)$ (the $N\times N$ matrix with entries $K(X_i,X_j)$) rather than the number of data points. To describe this write $\lambda_1 \geq \cdots \geq \lambda_r > 0$ for the nonzero eigenvalues of $K(X,X)$ indexed in decreasing order and write $\alpha_{\cdot,i}$ for the corresponding unit-normalized eigenvectors, i.e. $K(X,X)\alpha_{\cdot,i} = \lambda_i \alpha_{\cdot,i}$. Then $|f(X)|^2 = |f(\phi)|^2$, where $f(\phi)$ is the r vector with entries $f(\phi_i) := \sum_{s=1}^N f(X_s)\alpha_{s,i}$. Furthermore, writing $r' \leq r$ for the smallest index i such that $\lambda_i/\lambda_1 < \epsilon$ where $\epsilon > 0$ is some small threshold, the complexity of the problem can be further reduced (as in PCA) by truncating $f(\phi)$ to $f(\phi') = (f(\phi_1),\dots,f(\phi_{r'}))$ and approximating $\mathcal{F}$ with the space of functions $f \in \mathcal{H}$ such that $|f(\phi')|^2 \approx 0$.

D.2. Generalizing Descendants and Ancestors with Kernel Mode Decomposition.. We can extend the concept of descendants and ancestors to cover more complex functional dependencies between variables, including implicit ones. This generalization is achieved through a Kernel-based adaptation of Row Echelon Form Reduction (REFR), initially designed for affine systems, and leveraging the principles of Kernel Mode Decomposition (2). To describe the connection with REFR consider the example in which $\mathcal{M}$ is the manifold of $\mathbb{R}^3$ defined by the affine equations $x_1 + x_2 + 3x_3 - 2 = 0$ and $x_1 - x_2 + x_3 = 0$, which is equivalent to selecting $\mathcal{F} = \operatorname{span}\{f_1, f_2\}$ with $f_1(x) = x_1 + x_2 + 3x_3 - 2$ and $f_2(x) = x_1 - x_2 + x_3$ in the problem formulation 1. Then, irrespective of how we recover the manifold from data, the hypergraph representation of that manifold is equivalent to the row echelon form reduction of the affine system, and this representation and this reduction require a possibly arbitrary choice of free and dependent variables. So, for instance, if we declare x_3 to be the free variables and x_1 and x_2 to be the dependent variables, then we can represent the manifold via the equations $x_1 = 1 - 2x_3$ and $x_2 = 1 - x_3$ which have the hypergraph representation depicted in Fig. S6.(b). To describe the kernel generalization of REFR assume that the kernel K can be decomposed as the additive kernel

$$K = K_a + K_s + K_z\,, \tag{12}$$

and write $\mathcal{H}_a$, $\mathcal{H}_s$, and $\mathcal{H}_z$ for the RKHS induced by the kernels K_a, K_s, K_z. Then a function $f \in \mathcal{H}$ can be decomposed as $f = f_a + f_s + f_z$ with $(f_a, f_s, f_z) \in \mathcal{H}_a \times \mathcal{H}_s \times \mathcal{H}_z$. Then, generalizing REFR we can approximate the manifold $\mathcal{M}$ via a manifold parametrized by equations of the form

$$f_a + f_s + f_z = 0 \Leftrightarrow g_a = f_s \tag{13}$$

where $f_a = -g_a$ and g_a is a given function in $\mathcal{H}_a$ representing a dependent mode, $f_z = 0$ represents a zero mode, and $f_s \in \mathcal{H}_s$ is identified (regularized) as the minimizer of the following variational problem

$$\min_{f_s \in \mathcal{H}_s} \|f_s\|_{K_s}^2 + \frac{1}{\gamma}\left|(-g_a + f_s)(\phi)\right|^2 . \tag{14}$$

Taking $g_a(x) = x_1$ and $\mathcal{H}_s + \mathcal{H}_z$ to be a space of functions that does not depend on x_1 recovers our initial example Eq. (1)(with the pruning process encoded into the selection of $\mathcal{H}_z$). This generalization is motivated by its potential to recover implicit equations. For example, consider the implicit equation $x_1^2 + x_2^2 = 1$, which can be retrieved by setting the mode of interest to be $g_a(x) = x_1^2$ and allowing f_s to depend only on the variable x_2.

2. Algorithm Overview for Type 3 problems: An Informal Summary

In this section, we provide an accessible overview of our algorithm's key components, which are further detailed in Algorithms 1 and 2 in Section 6. Our method focuses on determining the edges within a hypergraph. To achieve this, we consider each node individually, finding its ancestors and establishing edges from these ancestors to the node in question. While we present the algorithm for a single node, it can be applied iteratively to all nodes within the graph.

Algorithm for finding the ancestors of a node:

1. **Initialization:** We start by assuming that all other nodes are potential ancestors of the current node.

2. **Selecting a Kernel**: We choose a kernel function, such as linear, quadratic, or fully nonlinear kernels (refer to Example 1). The kernel selection process is analogous to the subsequent pruning steps, involving the determination of a parameter γ, regression analysis, and evaluation based on signal-to-noise ratios.

   - **Kernel Selection Method:** The choice of kernel follows a process similar to the subsequent pruning steps, including γ selection, regression analysis, and signal-to-noise ratio evaluation.
   - **Low Signal-to-Noise Ratio for All Kernels:** If the signal-to-noise ratio is insufficient for all possible kernels, the algorithm terminates, indicating that the node has no ancestors.

3. **Pruning Process:** While there are potential ancestors left to consider (details in Section C.5):

   (a) **Identify the Least Important Ancestor:** Ancestors are ranked based on their contribution to the signal (see Sec. C.3).

(b) **Noise prior:** Determine the value of γ (see Section B).

(c) **Regression Analysis:** Predict the node's value using the current set of ancestors, excluding the least active one (i.e., the one contributing the least to the signal). We employ Kernel Ridge Regression with the selected kernel function and parameter γ (see Sec. C.3 and C.3).

(d) **Evaluate Removal:** Compute the regression signal-to-noise ratio (see Sec. C.4 and 7):

- **Low Signal-to-Noise Ratio:** If the signal-to-noise ratio falls below a certain threshold, terminate the algorithm and return the current set of ancestors (see Section C.6).
- **Adequate Signal-to-Noise Ratio:** If the signal-to-noise ratio is sufficient, remove the least active ancestor and continue the pruning process.

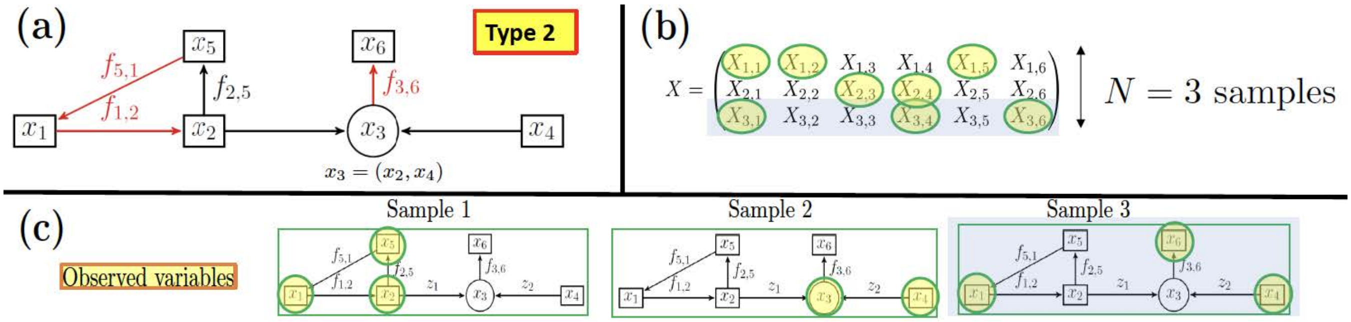

Fig. S2. Formal description of Type 2 problems.

3. Type 2 problems: Formal description and GP-based Computational Graph Completion

A. Formal description of Type 2 problems. Consider a computational graph (as illustrated in Fig. S2.(a)) where nodes represent variables and edges are directed and they represent functions. These functions may be known or unknown. In Fig. S2.(a), edges associated with unknown functions ($f_{5,1}$, $f_{1,2}$, $f_{3,6}$) are colored in red, and those associated with known functions ($f_{2,5}$) are colored in black. Round nodes are utilized to symbolize variables, which are derived from the concatenation of other variables (e.g, in Fig. S2.(a), $x_3 = (x_2, x_4)$). Therefore, the underlying graph is, in fact, a hypergraph where functions may map groups of variables to other groups of variables, and we use round nodes to illustrate the grouping step. Given partial observations derived from N samples of the graph's variables, we introduce a problem, termed a Type 2 problem, focused on approximating all unobserved variables and unknown functions. Using Fig. S2.(a)-(b) as an illustration we call a vector $(X_{s,1}, \dots, X_{s,6})$ a sample from the graph if its entries are variables satisfying the functional dependencies imposed by the structure of the graph (i.e., $X_{s,1} = f_{5,1}(X_{s,5})$, $X_{s,2} = f_{1,2}(X_{2,s})$, $X_{s,3} = (X_{s,2}, X_{s,4})$, $X_{s,5} = f_{s,5}(X_{s,s})$, and $X_{s,6} = f_{3,6}(X_{s,3})$. These samples can be seen as the rows of given matrix X illustrated in Fig. S2.(b) for $N = 3$. By partial observations, we mean that only a subset of the entries of each row may be observed, as illustrated in Fig. S2.(b)-(c). Note that a Type 2 problem combines a regression problem (approximating the unknown functions of the graph) with a matrix completion/data imputation problem (approximating the unobserved entries of the matrix X).

B. Reminder on Computational Graph Completion for Type 2 problems. Within the context of Sec. A, the proposed GP solution to Type 2 problems is to simply replace unknown functions by GPs and compute their Maximum A Posteriori (MAP)/Maximum Likelihood Estimation (MLE) estimators given available data and constraints imposed by the structure of the graph. Taking into account the example depicted in Fig. S2, and substituting $f_{5,1}$, $f_{1,2}$, and $f_{3,6}$ with independent GPs, each with kernels K, G, and Γ respectively, the objective of this MAP solution becomes minimizing $\|f_{5,1}\|_K^2 + \|f_{1,2}\|_G^2 + \|f_{3,6}\|_\Gamma^2$ (writing $\|f\|_K$ for the RKHS norm of f induced by the kernel K) subject to the constraints imposed by the data and the functional dependencies encoded into the structure of the graph.

C. A system identification example.. In order to exemplify Computational Graphical Completion (CGC), consider the system identification problem depicted in Fig. S3, sourced from (3). Our objective is to identify a nonlinear electric circuit, as illustrated in Fig. S3.(a), from scarce measurement data. The nonlinearity of the circuit emanates from the resistance, capacitance, and inductances, which are nonlinear functions of currents and voltages, as shown in Fig. S3.(b). Assuming these functions to be unknown, along with all currents and voltages as unknown time-dependent functions, we operate the circuit between times 0 and 10. Measurements of a subset of variables, representing the system's state, are taken at times $t_s = s/10$ for $s \in 0, \dots, 99$. Given these measurements, the challenge arises in approximating all unknown functions that define currents and voltages as time functions, capacitance as a voltage function, and inductances and resistance as current functions. Fig. S3.(c) displays the available measurements, which are notably sparse, preventing us from reconstructing the underlying unknown functions independently. Thus, their interdependencies must be utilized for approximation. It is crucial to note that the system's state variables are interconnected through functional relations, as per Kirchhoff's laws for this nonlinear electric circuit, illustrated in

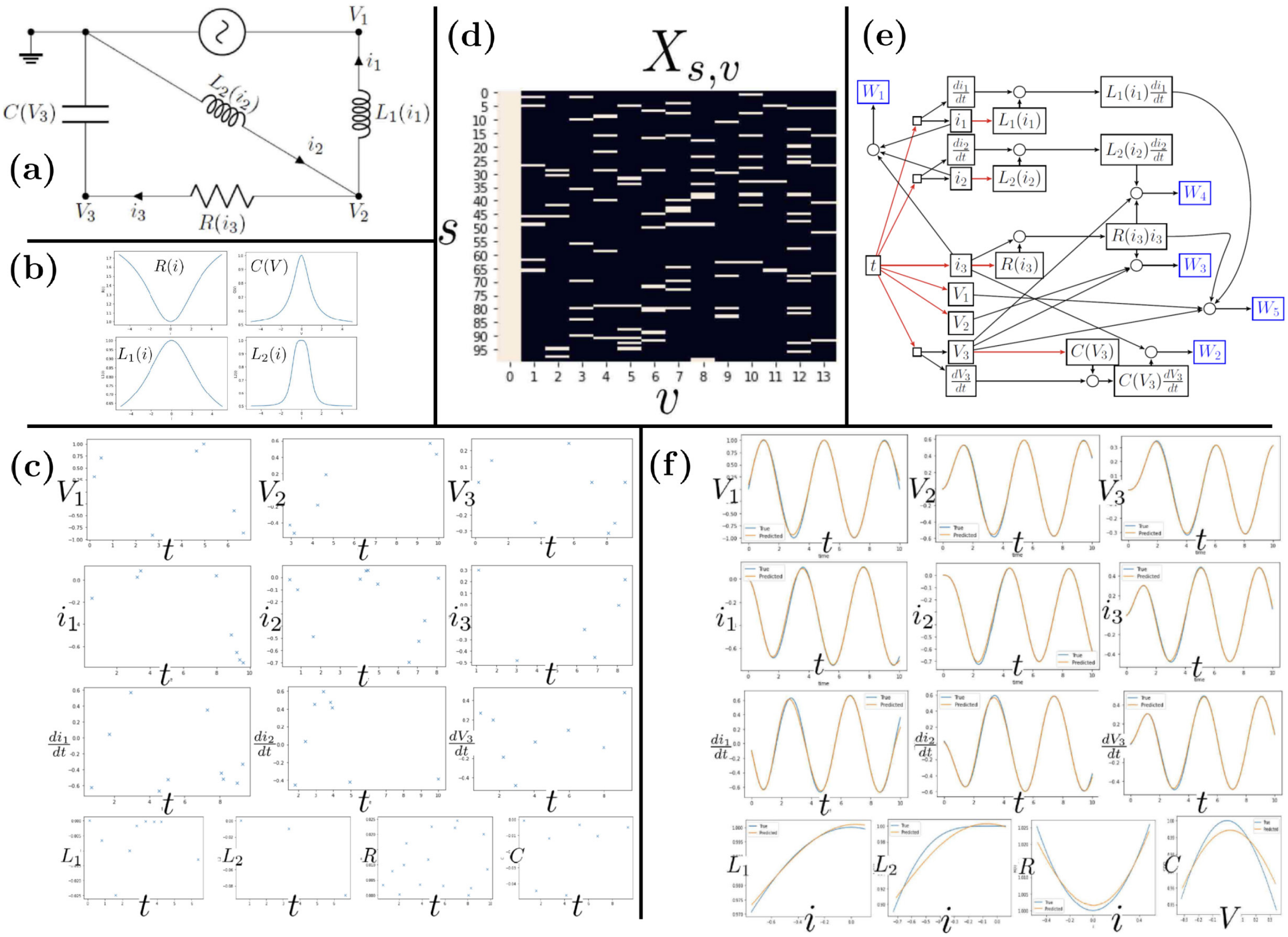

Fig. S3. (a) Electric circuit. (b) Resistance, capacitance, and inductances are nonlinear functions of currents and voltages (c) Measurements. (d) Kirchhoff's circuit laws. (e) The computational graph with unknown functions represented as red edges. (f) Recovered functions.

Fig. S3.(d). These functional dependencies can be conceptualized as a computational graph, depicted in Fig. S3.(e), where nodes represent variables and directed edges represent functions. Known functions are colored in black, unknown functions in red, and round nodes aggregate variables, meaning edges map groups of variables, forming a hypergraph. The CGC solution involves substituting the graph's unknown functions with Gaussian Processes (GPs), which may be independent or correlated, and then approximating the unknown functions with their Maximum A Posteriori (MAP) estimators, given the available data and the functional dependencies embedded in the graph's structure. Fig. S3.(f) showcases the true and recovered functions, demonstrating a notably accurate approximation despite the data's scarcity.

This simple example generalizes to an abstract framework detailed in (3). This framework has a wide range of applications because most problems in CSE can also be formulated as completing computational graphs representing dependencies between functions and variables, and they can be solved in a similar manner by replacing unknown functions with GPs and by computing their MAP/EB estimator given the data. These problems include those illustrated in Fig. 1.(d-h).

4. Hardness and well-posed formulation of Type 3 problems.

In this subsection, we describe why Type 3 problems are challenging and why they can even be intractable if not formalized and approached properly.

A. Curse of combinatorial complexity.. First, the problem suffers from the curse of combinatorial complexity in the sense that the number of hypergraphs associated with N nodes blows up rapidly with N. As an illustration, Fig. S4 shows some of the hypergraphs associated with only three nodes. A lower bound on that number is the A003180 sequence, which answers the following question (4): given N unlabeled vertices, how many different hypergraphs in total can be realized on them by counting the equivalent hypergraphs only once? For $N = 8$, this lower bound is $\approx 2.78 \times 10^{73}$.

B. Nonidentifiability and implicit dependencies.. Secondly, it is important to note that, even with an infinite amount of data, the exact structure of the hypergraph might not be identifiable. To illustrate this point, let's consider a problem where we have

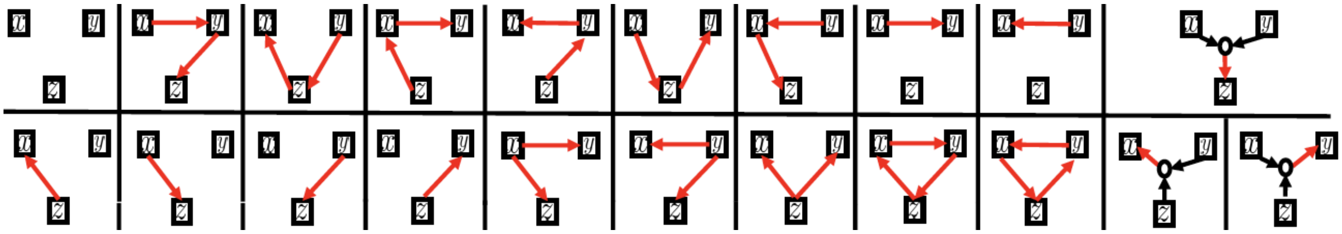

Fig. S4. Computational Hypergraph Discovery with three variables

N samples from a computational graph with variables x and y. The task is to determine the direction of functional dependency between x and y. Does it go from x to y (represented as $\boxed{x} \xrightarrow{f} \boxed{y}$), or from y to x (represented as $\boxed{y} \xrightarrow{f} \boxed{x}$)?

If we refer to Fig. S5.(a), we can make a decision because y can only be expressed as a function of x. In contrast, if we examine Fig. S5.(b), the decision is also straightforward because x can solely be written as a function of y. However, if the data mirrors the scenario in Fig. S5.(c), it becomes challenging to decide as we can write both y as a function of x and x as a function of y. Further complicating matters is the possibility of implicit dependencies between variables. As illustrated in Fig. S5.(d), there might be instances where neither y can be derived as a function of x, nor x can be represented as a function of y.

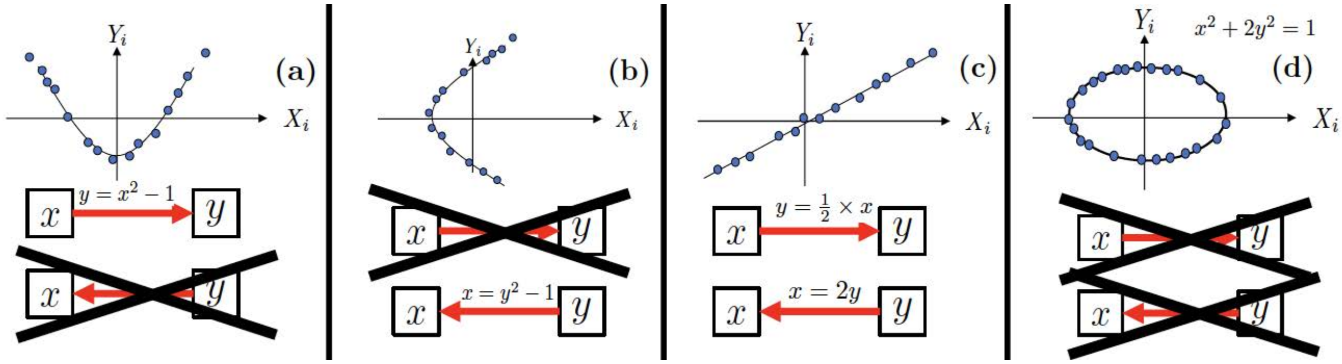

Fig. S5. The structure of the hypergraph is identifiable in (a), (b), and non-identifiable in (c). The relationship between variables is implicit in (d).

C. Causal inference and probabilistic graphs.. Causal inference methods broadly consist of two approaches: constraint and score-based methods. While constraint-based approaches are asymptotically consistent, they only learn the graph up to an equivalence class (5). Instead, score-based methods resolve ambiguities in the graph's edges by evaluating the likelihood of the observed data for each graphical model. For instance, they may assign a higher evidence to $y \to x$ over $x \to y$ if the conditional distribution $x|y$ exhibits less complexity than $y|x$. The complexity of searching over all possible graphs, however, grows super-exponentially with the number of variables. Thus, it is often necessary to use approximate, but more tractable, search-based methods (6, 7) or alternative criteria based on sensitivity analysis (8). For example, the preference could lean towards $y \to x$ rather than $x \to y$ if y demonstrates less sensitivity to errors or perturbations in x. In contrast, our proposed GP method avoids the growth in complexity by performing a guided pruning process that assesses the contribution of each node to the signal. We also emphasize that our method is not limited to learning acyclic graph structures as it can identify feedback loops between variables. Alternatively, methods for learning probabilistic undirected graphical models, also known as Markov networks, identify the graph structure by assuming the data is randomly drawn from some probability distribution (9). In this case, edges in the graph (or lack thereof) encode conditional dependencies between the nodes. A common approach learns the graph structure by modeling the data as being drawn from a multivariate Gaussian distribution with a sparse inverse covariance matrix, whose zero entries indicate pairwise conditional independencies (10). Recently, this approach has been extended using models for non-Gaussian distributions, e.g., in (11, 12), as well as kernel-based conditional independence tests (13). In this work, we learn functional dependencies rather than causality or probabilistic dependence. We emphasize that we also do not assume the data is randomized or impose strong assumptions, such as additive noise models, in the data-generating process.

We complete this paragraph by comparing the hypergraph discovery framework to structure learning for Bayesian networks and structural equation models (SEM). Let $x \in \mathbb{R}^d$ be a random variable with probability density function p that follows the autoregressive factorization $p(x) = \prod_{i=1}^{d} p_i(x_i|x_1, \ldots, x_{i-1})$ given a prescribed variable ordering. Structure learning for Bayesian networks aims to find the ancestors of variable x_i, often referred to as the set of parents $Pa(i) \subseteq \{1, \ldots, i-1\}$, in the sense that $p_i(x_i|x_1, \ldots, x_{i-1}) = p_i(x_i|x_{Pa(i)})$. Thus, the variable dependence of the conditional density p_i is identified by finding the parent set so that x_i is conditionally independent of all remaining preceding variables given its parents, i.e., $x_i \perp x_{1:i-1 \setminus Pa(i)}|x_{Pa(i)}$. Finding ancestors that satisfy this condition requires performing conditional independence tests, which are computationally

expensive for general distributions (14). Alternatively, SEMs assume that each variable x_i is drawn as a function of its ancestors with additive noise, i.e, $x_i = f(x_{Pa(i)}) + \epsilon_i$ for some function f and noise ϵ (7). For Gaussian noise $\epsilon_i \sim \mathcal{N}(0, \sigma^2)$, each marginal conditional distribution in a Bayesian network is given by $p_i(x_i|x_{1:i-1}) \propto \exp(-\frac{1}{2\sigma^2}\|x_i - f(x_{1:i-1})\|^2)$. Thus, finding the parents for such a model by maximum likelihood estimation corresponds to finding the parents that minimize the expected mean-squared error $\|x_i - f(x_{Pa(i)})\|^2$. Our approach minimizes a related objective, without imposing the strong probabilistic assumptions that are required in SEMs and Bayesian Networks. We also observe that while the graph structure identified in Bayesian networks is influenced by the specific sequence in which variables are arranged (a concept exploited in numerical linear algebra (15, 16) where Schur complementation is equivalent to conditioning GPs and a carefully ordering leads to the accuracy of the Vecchia approximation $p_i(x_i|x_1,\ldots,x_{i-1}) \approx p_i(x_i|x_{i-k},\ldots,x_{i-1})$ (17)), the graph recovered by our approach remains unaffected by any predetermined ordering of those variables.

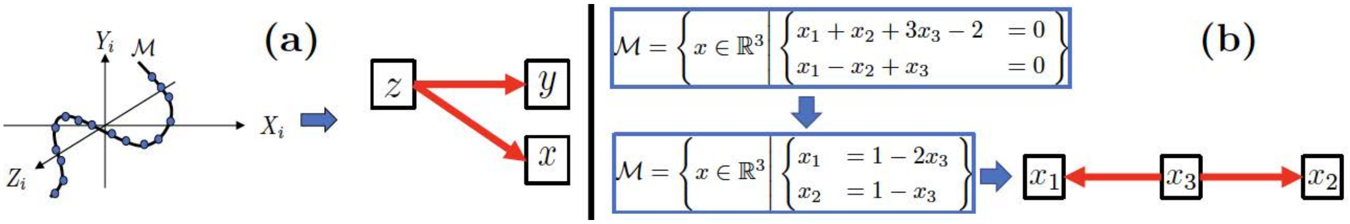

Fig. S6. (a) CHD formulation as a manifold discovery problem and hypergraph representation (b) The hypergraph representation of an affine manifold is equivalent to its Row Echelon Form Reduction.

D. Well-posed formulation of the problem.. In this paper, we focus on a formulation of the problem that remains well-posed even when the data is not randomized, i.e., we formulate the problem as the following manifold learning/discovery problem.

Problem 1. *Let $\mathcal{H}$ be a Reproducing Kernel Hilbert Space (RKHS) of functions mapping $\mathbb{R}^d$ to $\mathbb{R}$. Let $\mathcal{F}$ be a closed linear subspace of $\mathcal{H}$ and let $\mathcal{M}$ be a subset of $\mathbb{R}^d$ such that $x \in \mathcal{M}$ if and only if $f(x) = 0$ for all $f \in \mathcal{F}$. Given the (possibly noisy and nonrandom) observation of N elements, $X_1, \ldots, X_N$, of $\mathcal{M}$ approximate $\mathcal{M}$.*

To understand why problem 1 serves as the appropriate formulation for hypergraph discovery, consider a manifold $\mathcal{M} \subset \mathbb{R}^d$. Suppose this manifold can be represented by a set of equations, expressed as a collection of functions $(f_k)_k$ satisfying $\forall x \in \mathcal{M},\ f_k(x) = 0$. To keep the problem tractable, we assume a certain level of regularity for these functions, necessitating they belong to a RKHS $\mathcal{H}$, ensuring the applicability of kernel methods for our framework. Given that any linear combination of the f_k will also be evaluated to zero on $\mathcal{M}$, the relevant functions are those within the span of the f_k, forming a closed linear subspace of $\mathcal{H}$ denoted as $\mathcal{F}$. The manifold $\mathcal{M}$ can be subsequently represented by a graph or hypergraph (see Fig. S6.(a)), whose ambiguity can be resolved through a deliberate decision to classify some variables as free and others as dependent. This selection could be arbitrary, informed by expert knowledge, or derived from probabilistic models or sensitivity analysis.

5. A Gaussian Process method for Type 3 problems

A. Affine case and Row Echelon Form Reduction.. To describe the proposed solution to Problem 1, we start with a simple example. In this example $\mathcal{H}$ is a space of affine functions f of the form

$$f(x) = v^T\psi(x) \text{ with } \psi(x) := \begin{pmatrix} 1 \\ x \end{pmatrix} \text{ and } v \in \mathbb{R}^{d+1}\,,. \tag{15}$$

As a particular instantiation (see Fig. S6.(b)), we assume $\mathcal{M}$ to be the manifold of $\mathbb{R}^3$ ($d = 3$) defined by the affine equations

$$\mathcal{M} = \left\{ x \in \mathbb{R}^3 \,\middle|\, \begin{cases} x_1 + x_2 + 3x_3 - 2 &= 0 \\ x_1 - x_2 + x_3 &= 0 \end{cases} \right\}, \tag{16}$$

which is equivalent to selecting $\mathcal{F} = \text{span}\{f_1, f_2\}$ with $f_1(x) = x_1 + x_2 + 3x_3 - 2$ and $f_2(x) = x_1 - x_2 + x_3$ in the problem formulation 1.

Then, irrespective of how we recover the manifold from data, the hypergraph representation of that manifold is equivalent to the row echelon form reduction of the affine system, and this representation and this reduction require a possibly arbitrary choice of free and dependent variables. So, for instance, for the system Eq. (16), if we declare x_3 to be the free variables and x_1 and x_2 to be the dependent variables, then we can represent the manifold via the equations

$$\mathcal{M} = \left\{ x \in \mathbb{R}^3 \,\middle|\, \begin{cases} x_1 &= 1 - 2x_3 \\ x_2 &= 1 - x_3 \end{cases} \right\}, \tag{17}$$

which have the hypergraph representation depicted in Fig. S6.(b).

Now, in the $N > d$ regime where the number of data points is larger than the number of variables, the manifold can simply be approximated via a variant of PCA. Take $f^* \in \mathcal{F}$, we have $f^*(x) = v^{*T}\psi(x)$ for a certain $v^* \in \mathbb{R}^{d+1}$. Then for $X_s \in \mathcal{M}$, $f^*(X_s) = \psi(X_s)^T v^* = 0$. Defining

$$C_N := \sum_{s=1}^{N} \psi(X_s)\psi(X_s)^T \tag{18}$$

we see that $f^*(X_s) = 0$ for all X_s is equivalent to $C_N v^* = 0$. Since $N > d$, we can thus identify $\mathcal{F}$ exactly as $\{v^T\psi$ for $v \in Ker(C_N)\}$. We then obtain the manifold

$$\mathcal{M}_N = \left\{x \in \mathbb{R}^d \mid v^T\psi(x) = 0 \text{ for } v \in \text{Span}(v_{r+1}, \ldots, v_{d+1})\right\} \tag{19}$$

where $\text{Span}(v_{r+1}, \ldots, v_{d+1})$ is the zero-eigenspace of C_N. Here we write $\lambda_1 \geq \cdots \geq \lambda_r > 0 = \lambda_{r+1} = \cdots = \lambda_{d+1}$ for the eigenvalues of C_N (in decreasing order), and $v_1, \ldots, v_{d+1}$ for the corresponding eigenvectors ($C_N v_i = \lambda_i v_i$). The proposed approach extends to the noisy case (when the data points are perturbations of elements of the manifold) by simply replacing the zero-eigenspace of the covariance matrix by the linear span of the eigenvectors associated with eigenvalues that are smaller than some threshold $\epsilon > 0$, i.e., by approximating $\mathcal{M}$ with Eq. (19) where r is such that $\lambda_1 \geq \cdots \geq \lambda_r \geq \epsilon > \lambda_{r+1} \geq \cdots \geq \lambda_{d+1}$. In this affine setting Eq. (19) allows us to estimate $\mathcal{M}$ directly without RKHS norm minimization/regularization because linear regression does not require regularization in the sufficiently large data regime. Furthermore the process of pruning ancestors can be replaced by that of identifying sparse elements $v \in \text{Span}(v_{r+1}, \ldots, v_{d+1})$ such that $v_i = 1$.

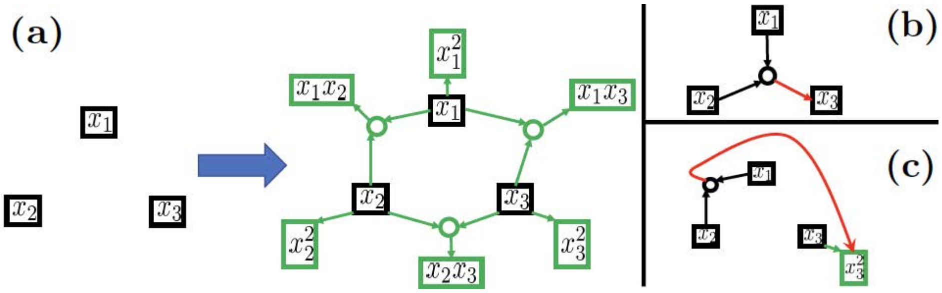

Fig. S7. Feature map generalization

B. Feature map generalization.. This simple approach can be generalized by generalizing the underlying feature map ψ used to define the space of functions (writing $d_{\mathcal{S}}$ for the dimension of the range of ψ)

$$\mathcal{H} = \left\{f(x) = v^T\psi(x) \mid v \in \mathbb{R}^{d_{\mathcal{S}}}\right\}. \tag{20}$$

For instance, if we use the feature map

$$\psi(x) := \left(1, \ldots, x_i, \ldots, x_i x_j, \ldots\right)^T \tag{21}$$

then $\mathcal{H}$ becomes a space of quadratic polynomials on $\mathbb{R}^d$, i.e.,

$$\mathcal{H} = \left\{f(x) = v_0 + \sum_i v_i x_i + \sum_{i \leq j} v_{i,j} x_i x_j \mid v \in \mathbb{R}^{d_{\mathcal{S}}}\right\}, \tag{22}$$

and, in the large data regime ($N > d_{\mathcal{S}}$), identifying quadratic dependencies between variables becomes equivalent to (1) adding nodes to the hypergraph corresponding to secondary variables obtained from primary variables x_i through known functions (for Eq. (21), these secondary variables are the quadratic monomials $x_i x_j$, see Fig. S7.(a)), and (2) identifying affine dependencies between the variables of the augmented hypergraph. The problem can, therefore, be reduced to the previous affine case. Indeed, as in the affine case, the manifold can then be approximated in the regime where the number of data points is larger than the dimension $d_{\mathcal{S}}$ of the feature map by Eq. (19), where $v_r, \ldots, v_N$ are the eigenvectors of C_N =Eq. (18) whose eigenvalues are zero (noiseless case) or smaller than some threshold $\epsilon > 0$ (noisy case).

Furthermore, the hypergraph representation of the manifold is equivalent to a feature map generalization of Row Echelon Form Reduction to nonlinear systems of equations. For instance, choosing x_3 as the dependent variable and x_1, x_2 as the free variables, $\mathcal{M} = \{x \in \mathbb{R}^3 \mid x_3 - 5x_1^2 + x_2^2 - x_1 x_2 = 0\}$ can be represented as in Fig. S7.(b) where the round node represents the concatenated variable (x_1, x_2) and the red arrow represents a quadratic function. The generalization also enables the representation of implicit equations by selecting secondary variables as free variables. For instance, selecting x_3^2 as the free variable and x_1, x_2 as the free variables, $\mathcal{M} = \{x \in \mathbb{R}^3 \mid x_1^2 + x_2^2 + x_3^2 - 1 = 0\}$ can be represented as in Fig. S7.(c).

C. Kernel generalization and regularization.. This feature-map extension of the previously discussed affine case can evidently be generalized to arbitrary degree polynomials and to other basis functions. However, as the dimension $d_{\mathcal{S}}$ of the range of the feature map ψ increases beyond the number N of data points, the problem becomes underdetermined: the data only provides partial information about the manifold, i.e., it is not sufficient to uniquely determine the manifold. Furthermore, if the dimension of the feature map is infinite, then we are always in that low data regime, and we have the additional difficulty that we cannot directly compute with that feature map. On the other hand, if $d_{\mathcal{S}}$ is finite (i.e., if the dictionary of basis functions is finite), then some elements of $\mathcal{F}$ (some constraints defining the manifold $\mathcal{M}$) may not be representable or well approximated as equations of the form $v^T\psi(x) = 0$. To address these conflicting requirements, we need to kernelize and regularize the proposed approach (as done in interpolation).

C.1. The kernel associated with the feature map.. To describe this kernelization, we assume that the feature map ψ maps $\mathbb{R}^d$ to some Hilbert space $\mathcal{S}$ that could be infinite-dimensional, and we write K for the kernel defined by that feature map. To be precise, we now consider the setting where the feature map ψ is a function from $\mathbb{R}^d$ to a (possibly infinite-dimensional separable) Hilbert (feature) space $\mathcal{S}$ endowed with the inner product $\langle\cdot,\cdot\rangle_{\mathcal{S}}$. To simplify notations, we will still write v^Tw for $\langle v, w\rangle_{\mathcal{S}}$ and vw^T for the linear operator mapping v' to $v\langle w, v'\rangle_{\mathcal{S}}$. Let

$$\mathcal{H} := \{v^T\psi(x) \mid v \in \mathcal{S}\} \qquad [23]$$

be the space of functions mapping $\mathbb{R}^d$ to $\mathbb{R}$ defined by the feature map ψ. To avoid ambiguity, assume (without loss of generality) that the identity $v^T\psi(x) = w^T\psi(x)$ holds for all $x \in \mathbb{R}^d$ if and only if $v = w$. It follows that for $f \in \mathcal{H}$ there exists a unique $v \in \mathcal{S}$ such that $f = v^T\psi$. For $f, g \in \mathcal{H}$ with $f = v^T\psi$ and $g = w^T\psi$, we can then define

$$\langle f, g\rangle_{\mathcal{H}} := v^Tw\,. \qquad [24]$$

Observe that $\mathcal{H}$ is a Hilbert space endowed with the inner product $\langle\cdot,\cdot\rangle_{\mathcal{H}}$. For $x, x' \in \mathcal{X}$, write

$$K(x, x') := \psi(x)^T\psi(x')\,, \qquad [25]$$

for the kernel defined by ψ and observe that $(\mathcal{H}, \langle\cdot,\cdot\rangle_{\mathcal{H}})$ is the RKHS defined by the kernel K (which is assumed to contain $\mathcal{F}$ in Problem 1). Observe in particular that for $f = v^T\psi \in \mathcal{H}$, K satisfies the reproducing property

$$\langle f, K(x,\cdot)\rangle_{\mathcal{H}} = v^T\psi(x) = f(x)\,. \qquad [26]$$

C.2. Complexity Reduction with Kernel PCA Variant.. We will now show that the previous feature-map PCA variant (characterizing the subspace of $f \in \mathcal{H}$ such that $f(X) = 0$) can be kernelized as a variant of kernel PCA (1). To describe this write $K(X, X)$ for the $N \times N$ matrix with entries $K(X_i, X_j)$. Write $\lambda_1 \geq \lambda_2 \geq \cdots \geq \lambda_r > 0$ for the nonzero eigenvalues of $K(X, X)$ indexed in decreasing order and write $\alpha_{\cdot,i}$ for the corresponding unit-normalized eigenvectors, i.e.

$$K(X, X)\alpha_{\cdot,i} = \lambda_i\alpha_{\cdot,i} \text{ and } |\alpha_{\cdot,i}| = 1\,. \qquad [27]$$

Write $f(X)$ for the N vector with entries $f(X_s)$. For $i \leq r$, write

$$\phi_i := \sum_{s=1}^{N} \delta_{X_s}\alpha_{s,i} \qquad [28]$$

and

$$f(\phi_i) := \sum_{s=1}^{N} f(X_s)\alpha_{s,i}\,. \qquad [29]$$

Write $f(\phi)$ for the r vector with entries $f(\phi_i)$.

Then, we have the following proposition.

Proposition 1. *The subspace of functions $f \in \mathcal{H}$ such that $f(\phi) = 0$ is equal to the subspace of $f \in \mathcal{H}$ such that $f(X) = 0$. Furthermore for $f \in \mathcal{H}$ with feature map representation $f = v^T\psi$ with $v \in \mathcal{S}$ we have the identity (where $C_N =$Eq. (18))*

$$v^TC_Nv = |f(\phi)|^2 = |f(X)|^2\,. \qquad [30]$$

Proof. Write $\hat{\lambda}_1 \geq \hat{\lambda}_2 \geq \cdots \geq \hat{\lambda}_{\hat{r}} > 0$ for the nonzero eigenvalues of $C_N =$Eq. (18) indexed in decreasing order. Write $v_1, \ldots, v_r$ for the corresponding eigenvectors, i.e.,

$$C_Nv_i = \hat{\lambda}_iv_i\,. \qquad [31]$$

Observing that

$$C_N = \sum_{i=1}^{r} \hat{\lambda}_iv_iv_i^T \qquad [32]$$

we deduce that the zero-eigenspace of C_N is the set of vectors $v \in \mathcal{S}$ such that $v^T v_i = 0$ for $i = 1, \ldots, r$. Write $f_i := v_i^T \psi$. Observe that for $f = v^T \psi$, we have $v_i^T v = \langle f_i, f \rangle_K$. Multiplying Eq. (31) by $\psi^T(x)$ implies

$$\sum_{s=1}^{N} K(x, X_s) f_i(X_s) = \hat{\lambda}_i f_i(x) \tag{33}$$

Eq. (33) implies that for $f = v^T \psi$

$$v_i^T v = \sum_{s=1}^{N} \hat{\lambda}_i^{-1} f_i(X_s) \langle K(\cdot, X_s), f \rangle_K = \sum_{s=1}^{N} \hat{\lambda}_i^{-1} f_i(X_s) f(X_s) \tag{34}$$

where we have used the reproducing property Eq. (26) of K in the last identity. Write

$$\hat{\alpha}_{s,i} := \lambda_i^{-1/2} f_i(X_s)\,. \tag{35}$$

Using Eq. (33) with $x = X_{s'}$ implies that $\hat{\alpha}_{\cdot,i}$ is an eigenvector of the $N \times N$ matrix $K(X, X)$ with eigenvalue $\hat{\lambda}_i$. Taking $f = f_i$ in Eq. (34) implies that $1 = v_i^T v_i = |\hat{\alpha}_{\cdot,i}|^2$. Therefore, the $\hat{\alpha}_{\cdot,i}$ are unit-normalized. Summarizing, this analysis (closely related to the one found in kernel PCA (1)) shows that the nonzero eigenvalues of $K(X, X)$ coincide with those of C_N and we have $\hat{r} = r$, $\hat{\lambda}_i = \lambda_i$ and $\hat{\alpha}_{\cdot,i} = \alpha_{\cdot,i}$. Furthermore, Eq. (34) and Eq. (35) imply that for $i \le r$, $v \in \mathcal{S}$ and $f = v^T \psi$, we have

$$v_i^T v = \lambda_i^{-1/2} f(X) \alpha_{\cdot,i}\,. \tag{36}$$

The identity Eq. (36) then implies Eq. (30). □

Remark 1. *As in PCA the dimension/complexity of the problem can be further reduced by truncating ϕ to $\phi' = (\phi_1, \ldots, \phi_{r'})$ where $r' \le r$ is identified as the smallest index i such that $\lambda_i/\lambda_1 < \epsilon$ where $\epsilon > 0$ is some small threshold.*

C.3. Kernel Mode Decomposition. When the feature map ψ is infinite-dimensional, the data only provides partial information about the constraints defining the manifold in the sense that $f(X) = 0$ or equivalently $f(\phi) = 0$ is a necessary but not sufficient condition for the zero level set of f to be a valid constraint for the manifold (for f to be such that $f(x) = 0$ for all $x \in \mathcal{M}$). So we are faced with the following problems: (1) How to regularize? (2) How do we identify free and dependent variables? (3) How do we identify valid constraints for the manifold? The proposed solution will be based on the Kernel Mode Decomposition (KMD) framework introduced in (2) (which shares conceptual foundations with Smoothing Spline ANOVA (18)).

Reminder on KMD We will now present a quick reminder on KMD in the setting of the following mode decomposition problem. So, in this problem, we have an unknown function $f^\dagger$ mapping some input space $\mathcal{X}$ to the real line $\mathbb{R}$. We assume that this function can be written as a sum of m other unknown functions $f_i^\dagger$ which we will call modes, i.e.,

$$f^\dagger = \sum_{i=1}^{m} f_i^\dagger\,. \tag{37}$$

We assume each mode $f_i^\dagger$ to be an unknown element of some RKHS $\mathcal{H}_{K_i}$ defined by some kernel K_i. Then we consider the problem in which given the data $f^\dagger(X) = Y$ (with $(X, Y) \in \mathcal{X}^N \times \mathbb{R}^N$) we seek to approximate the m modes composing the target function $f^\dagger$. Then, we have the following theorem.

Theorem 1. *(2) Using the relative error in the product norm $\|(f_1, \ldots, f_m)\|^2 := \sum_{i=1}^m \|f_i\|_{K_i}^2$ as a loss, the minimax optimal recovery of $(f_1^\dagger, \ldots, f_m^\dagger)$ is $(f_1, \ldots, f_m)$ with*

$$f_i(x) = K_i(x, X) K(X, X)^{-1} Y\,,, \tag{38}$$

where K is the additive kernel

$$K = \sum_{i=1}^{m} K_i\,. \tag{39}$$

The GP interpretation of this optimal recovery result is as follows. Let $\xi_i \sim \mathcal{N}(0, K_i)$ be m independent centered GPs with kernels K_i. Write ξ for the additive GP $\xi := \sum_{i=1}^m \xi_i$. Eq. (38) can be recovered by replacing the modes $f_i^\dagger$ by independent centered GPs $\xi_i \sim \mathcal{N}(0, K_i)$ with kernels K_i and approximating the mode i by conditioning ξ_i on the available data $\xi(X) = Y$ where $\xi := \sum_{i=1}^m \xi_i$ is the additive GP obtained by summing the independent GPs ξ_i, i.e.,

$$f_i(x) = \mathbb{E}\big[\xi_i(x) \mid \xi(X) = Y\big]\,. \tag{40}$$

Furthermore $(f_1, \ldots, f_m)$ can also be identified as the minimizer of

$$\begin{cases} \text{Minimize} & \sum_{i=1}^m \|f_i\|_{K_i}^2 \\ \text{over} & (f_1, \ldots, f_m) \in \mathcal{H}_{K_1} \times \cdots \times \mathcal{H}_{K_m} \\ \text{s. t.} & (\sum_{i=1}^m f_i)(X) = Y\,. \end{cases} \tag{41}$$

The variational formulation Eq. (41) can be interpreted as a generalization of Tikhonov regularization which can be recovered by selecting $m = 2$, K_1 to be a smoothing kernel (such as a Matérn kernel) and $K_2(x, y) = \sigma^2\delta(x - y)$ to be a white noise kernel.

Now, this abstract KMD approach (2) is associated with a quantification of how much each mode contributes to the overall data or how much each individual GP ξ_i explains the data. More precisely, the activation of the mode i or GP ξ_i can be quantified as

$$p(i) = \frac{\|f_i\|_{K_i}^2}{\|f\|_K^2}, \qquad [42]$$

where $f = \sum_{i=1}^m f_i$. These activations $p(i)$ satisfy $p(i) \in [0, 1]$ and $\sum_{i=1}^m p(i) = 1$ they can be thought of as a generalization of Sobol sensitivity indices (19–21) to the nonlinear setting in the sense that they are associated with the following variance representation/decomposition (2) (writing $\langle\cdot,\cdot\rangle_K$ for the RKHS inner product induced by K):

$$\mathrm{Var}\left[\langle\xi, f\rangle_K\right] = \|f\|_K^2 = \sum_{i=1}^m \|f_i\|_{K_i}^2 = \sum_{i=1}^m \mathrm{Var}\left[\langle\xi_i, f\rangle_K\right] \qquad [43]$$

Application to CHD, general case. Now, let us return to our original manifold approximation problem 1 in the kernelized setting of Eq. (25). Given the data X we cannot regress an element $f \in \mathcal{F}$ directly since the minimizer of $\|f\|_K^2 + \gamma^{-1}\|f(X)\|_{\mathbb{R}^N}^2$ is the null function. To identify the functions $f \in \mathcal{F}$, we need to decompose them into modes that can be interpreted as a generalization of the notion of free and dependent variables. To describe this, assume that the kernel K can be decomposed as the additive kernel

$$K = K_a + K_s + K_z. \qquad [44]$$

Then $\mathcal{H}_K = \mathcal{H}_{K_a} + \mathcal{H}_{K_s} + \mathcal{H}_{K_z}$ implies that for all function $f \in \mathcal{H}_K$, f can be decomposed as $f = f_a + f_s + f_z$ with $(f_a, f_s, f_z) \in \mathcal{H}_a \times \mathcal{H}_s \times \mathcal{H}_z$.

Example 1. *As a running example, take K to be the following additive kernel*

$$K(x, x') = 1 + \beta_1 \sum_i x_i x_i' + \beta_2 \sum_{i \le j} x_i x_j x_i' x_j' + \beta_3 \prod_i (1 + k(x_i, x_i')), \qquad [45]$$

that is the sum of a linear kernel, a quadratic kernel, and a fully nonlinear kernel. Take K_a to be the part of the linear kernel that depends only on x_1, i.e.,

$$K_a(x, x') = \beta_1 x_1 x_1'. \qquad [46]$$

Take K_s to be the part of the kernel that does not depend on x_1, i.e.,

$$K_s = 1 + \beta_1 \sum_{i \ne 1} x_i x_i' + \beta_2 \sum_{i \le j, i, j \ne 1} x_i x_j x_i' x_j' + \beta_3 \prod_{i \ne 1} (1 + k(x_i, x_i')). \qquad [47]$$

And take K_z to be the remaining portion,

$$K_z = K - K_a - K_s. \qquad [48]$$

Therefore the following questions are equivalent:

- Given a function g_a in the RKHS $\mathcal{H}_{K_a}$ defined by the kernel K_a is there a function f_s in the RKHS $\mathcal{H}_{K_s}$ defined by the kernel K_s such that $g_a(x) \approx f_s(x)$ for $x \in \mathcal{M}$?
- Given a function $g_a \in \mathcal{H}_{K_a}$ is there a function f in the RKHS $\mathcal{H}_K$ defined by the kernel K such that $f(x) \approx 0$ for $x \in \mathcal{M}$ and such that its f_a mode is $-g_a$ and its f_z mode is zero?

Then, the natural answer to the questions is to identify the modes of the constraint $f = f_a + f_s + f_z \in \mathcal{H}$ (such that $f(x) \approx 0$ for $x \in \mathcal{M}$) such that $f_a = -g_a$ and $f_z = 0$ by selecting f_s to be the minimizer of the following variational problem

$$\min_{f_s \in \mathcal{H}_s} \|f_s\|_{K_s}^2 + \frac{1}{\gamma}\left|(-g_a + f_s)(\phi)\right|^2. \qquad [49]$$

This is equivalent to introducing the additive GP $\xi = \xi_a + \xi_s + \xi_z + \xi_n$ whose modes are the independent GPs $\xi_a \sim \mathcal{N}(0, K_a)$, $\xi_s \sim \mathcal{N}(0, K_s)$, $\xi_z \sim \mathcal{N}(0, K_z)$, $\xi_n \sim \mathcal{N}(0, \gamma\delta(x - y))$ (we use the label "n" in reference to "noise"), and then recovering f_s as

$$f_s = \mathbb{E}\left[\xi_s \mid \xi(X) = 0, \xi_a = -g_a, \xi_z = 0\right]. \qquad [50]$$

Application to CHD, particular case. Taking $g_a(x) = x_1$ for our running example 1, the previous questions are, as illustrated in Fig. 2.(b), equivalent to asking whether there exists a function $f_s \in \mathcal{H}_{K_s}$ that does not depend on x_1 (since K_s does not depend on x_1) such that

$$x_1 \approx f_s(x_2, \ldots, x_d) \text{ for } x \in \mathcal{M}. \qquad [51]$$

Therefore, the mode f_a can be thought of as a dependent mode (we use the label "a" in reference to "ancestors"), the mode f_s as a free mode (we use the label "s" in reference to "signal"), the mode f_z as a zero mode.

While our numerical illustrations have primarily focused on the scenario where g_a takes the form of $g_a(x) = x_i$, and we aim to express x_i as a function of other variables, the generality of our framework is motivated by its potential to recover implicit equations. For example, consider the implicit equation $x_1^2 + x_2^2 = 1$, which can be retrieved by setting the mode of interest to be $g_a(x) = x_1^2$ and allowing f_s to depend only on the variable x_2.

C.4. Signal-to-noise ratio.. Now, we are led to the following question: since the mode f_s (the minimizer of Eq. (49)) always exists and is always unique, how do we know that it leads to a valid constraint? To answer that question, we compute the activation of the GPs used to regress the data. We write

$$\mathcal{V}(s) := \|f_s\|_{K_s}^2 \,, \qquad [52]$$

for the activation of the signal GP ξ_s and

$$\mathcal{V}(n) := \frac{1}{\gamma}\left|(-g_a + f_s)(X)\right|^2 \qquad [53]$$

for the activation of the noise GP ξ_n, and then these allow us to define a signal-to-noise ratio defined as

$$\frac{\mathcal{V}(s)}{\mathcal{V}(s)+\mathcal{V}(n)} \,. \qquad [54]$$

Note that this corresponds to activation ratio of the noise GP defined in (42). This ratio can then be used to test the validity of the constraint in the sense that if $V(s)/(V(s)+V(n)) > \tau$ (with $\tau = 0.5$ as a prototypical example), then the data is mostly explained by the signal GP and the constraint is valid. If $V(s)/(V(s)+V(n)) < \tau$, then the data is mostly explained by the noise GP and the constraint is not valid.

C.5. Iterating by removing the least active modes from the signal.. If the constraint is valid, then we can next compute the activation of the modes composing the signal. To describe this, we assume that the kernel K_s can be decomposed as the additive kernel

$$K_s = K_{s,1} + \cdots + K_{s,m} \,, \qquad [55]$$

which results in $\mathcal{H}_{K_s} = \mathcal{H}_{K_{s,1}} + \cdots + \mathcal{H}_{K_{s,m}}$, which results in the fact that $\forall f_s \in \mathcal{H}_s$, f_s can be decomposed as

$$f_s = f_{s,1} + \cdots + f_{s,m} \,, \qquad [56]$$

with $f_{s,i} \in \mathcal{H}_{K_{s,i}}$. The activation of the mode i can then be quantified as $p(i) = \|f_{s,i}\|_{K_{s,i}}^2 / \|f_s\|_{K_s}^2$, which combined with $\|f_s\|_{K_s}^2 = \sum_{i=1}^m \|f_{s,i}\|_{K_{s,i}}^2$ leads to $\sum_{i=1}^m p(i) = 1$.

As our running example 1, we can decompose K_s =Eq. (47) as the sum of an affine kernel, a quadratic kernel, and a fully nonlinear kernel, i.e., $m = 3$, $K_{s,1} = 1 + \beta_1 \sum_{i\neq 1} x_i x_i'$, $K_{s,2} = \beta_2 \sum_{i\leq j, i, j\neq 1} x_i x_j x_i' x_j'$ and $K_{s,3} = \beta_3 \prod_{i\neq 1}(1 + k(x_i, x_i'))$.

As another example for our running example, we can take K_s to be the sum of the portion of the kernel that does not depend on x_1 and x_2 and the remaining portion, i.e., $m = 2$, $K_{s,1} = 1 + \beta_1 \sum_{i\neq 1,2} x_i x_i' + \beta_2 \sum_{i\leq j, i, j\neq 1,2} x_i x_j x_i' x_j' + \beta_3 \prod_{i\neq 1,2}(1 + k(x_i, x_i'))$ and $K_{s,2} = K_s - K_{s,1}$.

Then, we can order these sub-modes from most active to least active and create a new kernel K_s by removing the least active modes from the signal and adding them to the mode that is set to be zero (see Fig. S8). To describe this, let $\pi(1), \cdots, \pi(m)$ be an ordering of the modes by their activation, i.e., $\|f_{s,\pi(1)}\|_{K_{s,\pi(1)}}^2 \geq \|f_{s,\pi(2)}\|_{K_{s,\pi(2)}}^2 \geq \cdots$.

Writing $K_t = \sum_{i=r+1}^m K_{s,\pi(i)}$ for the additive kernel obtained from the least active modes (with $r + 1 = m$ as the value used for our numerical implementations), we update the kernels K_s and K_z by assigning the least active modes from K_s to K_z, i.e., $K_s - K_t \to K_s$ and $K_z + K_t \to K_z$ (we zero the least active modes).

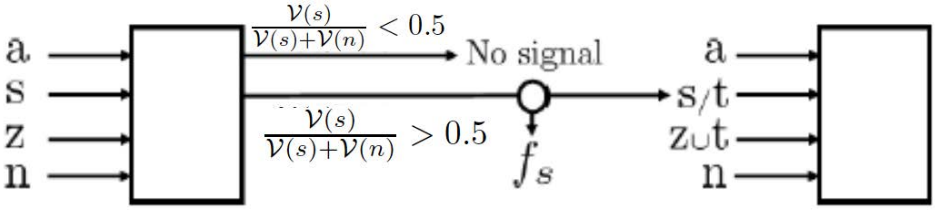

Fig. S8. Iterating by removing the least active modes from the signal

Finally, we can iterate the process. This iteration can be thought of as identifying the structure of the hypergraph by placing too many hyperedges and removing them according to the activation of the underlying GPs.

For our running example 1, where we try to identify the ancestors of the variable x_1, if the sub-mode associated with the variable x_2 is found to be least active, then we can try to remove x_2 from the list of ancestors and try to identify x_1 as a function of x_3 to x_d. This is equivalent to selecting $K_a(x, x') = \beta_1 x_1 x_1'$,

$$K_{s/t} = 1 + \beta_1 \sum_{i\neq 1,2} x_i x_i' + \beta_2 \sum_{i\leq j, i, j\neq 1,2} x_i x_j x_i' x_j' + \beta_3 \prod_{i\neq 1,2} (1 + k(x_i, x_i')) \,, \qquad [57]$$

and $K_{z\cup t} = K - K_a - K_{s/t}$ to assess whether there exists a function $f_s \in \mathcal{H}_K$ that does not depend on x_1 and x_2 s.t. $x_1 \approx f_s(x_3, \ldots, x_d)$ for $x \in \mathcal{M}$.

C.6. Alternative determination of the list of ancestors.. Our initial approach to determining the list of ancestors of a given node is to use a fixed threshold (e.g., $\tau = 0.5$) to prune nodes. We propose a refined approach that mimics the strategy employed in Principal Component Analysis (PCA) for deciding which modes should be kept and which ones should be removed. The PCA approach is to order the modes in decreasing order of eigenvalues/variance and (1) either keep the smallest number modes holding/explaining a given fraction (e.g., 90%) of the variance in the data, (2) or use an inflection point/sharp drop in the decay of the eigenvalues to select which modes should be kept. Here, we propose a similar strategy. First we employ an alternative determination of the least active mode: we iteratively remove the mode that leads to the smallest increase in noise-to-signal ratio, i.e., we remove the mode t such that,

$$t = \operatorname{argmin}_t \frac{\mathcal{V}(n)}{\mathcal{V}(s/t) + \mathcal{V}(n)} . \tag{58}$$

For our running example 1 in which we try to find the ancestors of the variable x_1 this is equivalent to removing the variables or node t whose removal leads to the smallest loss in signal-to-noise ratio (or increase in noise-to-signal ratio) by selecting

$$K_{s/t} = 1 + \beta_1 \sum_{i \neq 1,t} x_i x_i' + \beta_2 \sum_{i \leq j, i, j \neq 1,t} x_i x_j x_i' x_j' + \beta_3 \prod_{i \neq 1,t} (1 + k(x_i, x_i')) .$$

Next, we iterate this process, and we plot (a) the noise-to-signal ratio, and (b) the increase in noise-to-signal ratio as a function of the number of ancestors ordered according to this iteration. Fig. S9 illustrates this process and shows that the removal of an essential node leads to a sharp spike in increase in the noise-to-signal ratio (the noise-to-signal ratio jumps from approximately 50-60% to 99%). The identification of this inflection point can be used as a method for effectively and reliably pruning ancestors.

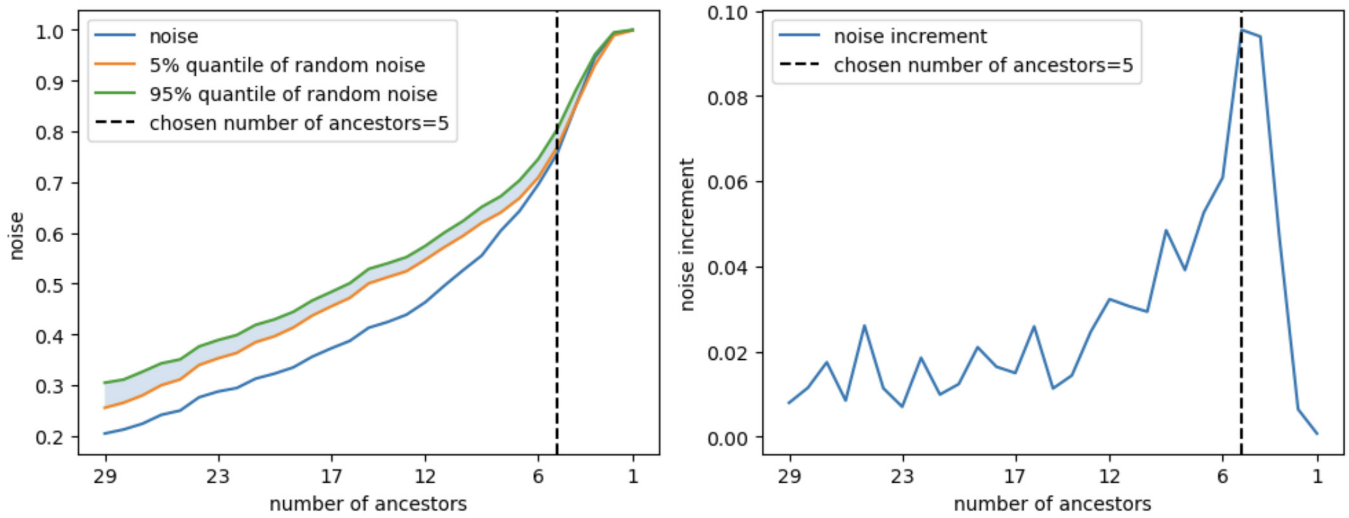

Fig. S9. Computing the ancestors of the variable $\dot{x}_0$ in the Fermi-Pasta-Ulam-Tsingou problem. (a) Noise-to-Signal Ratio, denoted as $\frac{\mathcal{V}(n)}{\mathcal{V}(s)+\mathcal{V}(n)}(q)$, with respect to the number of proposed ancestors, represented by q. Additionally, we include a visualization of the quantiles derived from the Z-test, as described in Section C. Notably, when there is no signal present, the noise-to-signal ratio is expected to fall within the shaded area with a probability of 0.9. (b) Increments in the Noise-to-Signal Ratio, defined as $\frac{\mathcal{V}(n)}{\mathcal{V}(s)+\mathcal{V}(n)}(q) - \frac{\mathcal{V}(n)}{\mathcal{V}(s)+\mathcal{V}(n)}(q-1)$, as a function of the number of ancestors, denoted as q. The horizontal axis represents the number of proposed ancestors for $\dot{x}_0$. Determining an appropriate stopping point based solely on absolute noise-to-signal ratio levels can be challenging. In contrast, the increments in the noise-to-signal ratio clearly exhibit a discernible maximum, offering a practical point for decision-making.

6. Algorithm pseudocode.

Our overall method is summarized in the pseudocode Alg. 1 and Alg. 2 that we will now describe. Alg. 1 takes the data D (encoded into the samples $X_1, \ldots, X_N$ of Problem 1) and the set of nodes V as an input and produces, as described in Sec. C, for each node $i \in V$ its set of minimal ancestors A_i and the simplest possible function f_i such that $x_i \approx f_i\big((x_j)_{j \in A_i}\big)$. It employs the default threshold of 0.5 on the signal-to-noise ratios for its operations. Line 1 normalizes the data (via an affine transformation) so that the samples X_i are of mean zero and variance 1. Given a node with index $i = 1$ in Line 2 (i runs through the set of nodes, and we select $i = 1$ for ease of presentation), the command in Line 3 refers to selecting a signal kernel of the form K_s =Eq. (47) (where k is selected to be a vanilla RBF kernel such as Gaussian or Matérn), with $1 \geq \beta_1 > 0 = \beta_2 = \beta_3$ for the linear kernel, $1 \geq \beta_1 \geq \beta_2 > 0 = \beta_3$ for the quadratic kernel and $1 \geq \beta_1 \geq \beta_2 \geq \beta_3 > 0$ for the fully nonlinear (interpolative) kernel. The ComputeSignalToNoiseRatio function in Line 5 computes the signal-to-noise ratio with $g_a(x) = x_1$ and with the kernel selected in Line 3. The value of γ is selected automatically by maximizing the variance of the histogram of eigenvalues of D_γ as described in Sec. B (with the kernel $K = K_s$ =Eq. (47) selected in Line 3 and $Y = g_a(X)$ with $g_a(x) = x_1$). The value of γ is re-computed whenever a node is removed from the list of ancestors, and K_s is nonlinear. Lines 9, 10 and 11 are described in Sec. C.5. They correspond to iteratively identifying the ancestor node t contributing the

Algorithm 1 CHD by thresholding the signal-to-noise ratio

Input: Data D, set of nodes V, threshold τ ($\tau = 0.5$ as a default value)
Output: Learned hypergraph // Set of ancestors for each node
$D \leftarrow$ NormalizeData(D) // Normalize the data
for $v \in V$ **do**
  for kernel $\in$ ["linear", "quadratic", "nonlinear"] **do** // Find the kernel
    SetOfAncestors(v) $\leftarrow$ All other nodes
    SignalToNoiseRatio $\leftarrow$ ComputeSignalToNoiseRatio(kernel, node, D)
    if SignalToNoiseRatio $> \tau$ **then** choose that kernel and exit the for loop
    else remove all ancestors from node
  while SignalToNoiseRatio $> \tau$ **do** // Prune ancestors
    Find least important ancestor
    Recompute SignalToNoiseRatio without ancestor
    if SignalToNoiseRatio $> \tau$ **then** Remove that ancestor

least to the signal and removing that node from the set of ancestors of the node 1 if the removal of that node t does not send the signal-to-noise ratio below the default threshold 0.5.

Algorithm 2 CHD by inflection point in the noise-to-signal ratio

Input: Data D, set of nodes V, threshold τ ($\tau = 0.5$ as a default value)
Output: Learned hypergraph // Set of ancestors for each node
$D \leftarrow$ NormalizeData(D) // Normalize the data
for node $v \in V$ **do**
  for kernel $\in$ ["linear", "quadratic", "nonlinear"] **do** // Find the kernel
    SetOfAncestors $\leftarrow$ All other nodes
    SignalToNoiseRatio $\leftarrow$ ComputeSignalToNoiseRatio(kernel, node, D)
    if SignalToNoiseRatio $> \tau$ **then** choose that kernel and exit the for loop
    else remove all ancestors from node
  q $\leftarrow$ Cardinal(All other nodes)
  SetOfAncestors(q) $\leftarrow$ All other nodes
  while q ≥ 1 **do**
    NoiseToSignalRatio(q) $\leftarrow$ ComputeNoiseToSignalRatio(kernel, node, D)
    LeastImportantAncestor $\leftarrow$ Find least important ancestor in SetOfAncestors(q)
    SetOfAncestors($q-1$) $\leftarrow$ SetOfAncestors(q) $\setminus$ LeastImportantAncestor
    $q \leftarrow q - 1$
  $q^\dagger \leftarrow$ Inflection point in ($q \rightarrow$ NoiseToSignalRatio(q)) or spike in ($q \rightarrow$ NoiseToSignalRatio(q) - NoiseToSignalRatio($q-1$))
  FinalSetOfAncestors(v) $\leftarrow$ SetOfAncestors($q^\dagger$)

Algorithm 2 distinguishes itself from Algorithm 1 in its approach to pruning ancestors based on signal-to-noise ratios. Instead of using a default threshold of 0.5 like Algorithm 1, Algorithm 2 computes the noise-to-signal ratio, represented as $\frac{\mathcal{V}(n)}{\mathcal{V}(s)+\mathcal{V}(n)}(q)$. This ratio is calculated as a function of the number q of ancestors, which are ordered based on their decreasing contribution to the signal. The detailed methodology behind this computation can be found in Section C.6 and is visually depicted in Figure S9. The final number q of ancestors is then determined by finding the value that maximizes the difference between successive noise-to-signal ratios, $\frac{\mathcal{V}(n)}{\mathcal{V}(s)+\mathcal{V}(n)}(q+1) - \frac{\mathcal{V}(n)}{\mathcal{V}(s)+\mathcal{V}(n)}(q)$.

7. Analysis of the signal-to-noise ratio test.

A. The signal-to-noise ratio depends on the prior on the level of noise.. The signal-to-noise ratio Eq. (54) depends on the value of γ, which is the variance prior on the level of noise. The goal of this subsection is to answer the following two questions: (1) How do we select γ? (2) How do we obtain a confidence level for the presence of a signal? Or equivalently for a hyperedge of the hypergraph? To answer these questions, we will now analyze the signal-to-noise ratio in the following regression problem in which we seek to approximate the unknown function $f^\dagger : \mathcal{X} \rightarrow \mathbb{R}$ based on noisy observations

$$f^\dagger(X) + \sigma Z = Y \tag{59}$$

of its values at collocation points X_i ($(X, Y) \in \mathcal{X}^N \times \mathbb{R}^N$, $Z \in \mathbb{R}^N$, and the entries Z_i of Z are i.i.d $\mathcal{N}(0,1)$). Assuming σ^2 to be unknown and writing γ for a candidate for its value, recall that the GP solution to this problem is approximate $f^\dagger$ by interpolating the data with the sum of two independent GPs, i.e.,

$$f(x) = \mathbb{E}[\xi(x)|\xi(X) + \sqrt{\gamma}Z = Y], \tag{60}$$

where $\xi \sim \mathcal{N}(0, K)$ is the GP prior for the signal $f^\dagger$ and $\sqrt{\gamma}Z \sim \mathcal{N}(0, \gamma I_N)$ is the GP prior for the noise σZ in the measurements. Following Sec. C.3 f can also be identified as a minimizer of

$$\text{minimize}_{f'} \|f'\|_K^2 + \frac{1}{\gamma}\|f'(X) - Y\|_{\mathbb{R}^N}^2 \,, \tag{61}$$

the activation of the signal GP can be quantified as $s = \|f\|_K^2$, the activation of the noise GP can be quantified as $\mathcal{V}(n) = \frac{1}{\gamma}\|f(X) - Y\|_{\mathbb{R}^N}^2$. We can then define the noise to signal ratio $\frac{\mathcal{V}(n)}{\mathcal{V}(s)+\mathcal{V}(n)}$, which admits the following representer formula,

$$\frac{\mathcal{V}(n)}{\mathcal{V}(s)+\mathcal{V}(n)} = \gamma \frac{Y^T\big(K(X,X)+\gamma I\big)^{-2}Y}{Y^T\big(K(X,X)+\gamma I\big)^{-1}Y} \,. \tag{62}$$

Observe that when applied to the setting of Sec. C.4, this signal-to-noise ratio is calculated with $K = K_s$ and $Y = g_a(X)$.

Now we have the following proposition, which follows from Eq. (62).

Proposition 2. *It holds true that* $\frac{\mathcal{V}(n)}{\mathcal{V}(s)+\mathcal{V}(n)} \in [0, 1]$, *and if* $K(X, X)$ *has full rank,*

$$\lim_{\gamma\downarrow 0} \frac{\mathcal{V}(n)}{\mathcal{V}(s)+\mathcal{V}(n)} = 0 \textit{ and } \lim_{\gamma\uparrow\infty} \frac{\mathcal{V}(n)}{\mathcal{V}(s)+\mathcal{V}(n)} = 1 \,. \tag{63}$$

Therefore, we are led to the following question: if the signal $f^\dagger$ and the level of noise σ^2 are both unknown, how do we select γ to decide whether the data is mostly signal or noise?

B. How do we select the prior on the level of noise?. Our answer to this question depends on whether the feature-map associated with the base kernel K is finite-dimensional or not.

B.1. When the kernel is linear, quadratic or associated with a finite-dimensional feature map.. If the feature-map associated with the base kernel K is finite-dimensional, then γ can be estimated from the data itself when the number of data-points is sufficiently large (at least larger than the dimension of the feature-space $\mathcal{S}$). A prototypical example (when trying to identify the ancestors of the variable x_1) is $K = K_s$=Eq. (47) with $\beta_3 = 0$. In the general setting assume that $K(x, x') := \psi(x)^T\psi(x')$ where the range $\mathcal{S}$ of ψ is finite-dimensional. Assume that $f^\dagger$ belongs to the RKHS defined by ψ, i.e., assume that it is of the form $f^\dagger = v^T\psi$ for some v in the feature-space. Then Eq. (59) reduces to

$$v^T\psi(X) + \sigma Z = Y \,, \tag{64}$$

and, in the large data regime, σ^2 can be estimated by

$$\bar{\sigma}^2 := \frac{1}{N}\inf_{w\in\mathcal{S}} \left\|w^T\psi(X) - Y\right\|_{\mathbb{R}^N}^2 \,. \tag{65}$$

Our strategy, when the feature map is finite-dimensional, is then to select

$$\gamma = N\bar{\sigma}^2 = \inf_{w\in\mathcal{S}} \left\|w^T\psi(X) - Y\right\|_{\mathbb{R}^N}^2 \,. \tag{66}$$

B.2. When the kernel is interpolatory (associated with an infinite-dimensional feature map).. If the feature-map associated with the base kernel K is infinite-dimensional (or has more dimensions than we have data points) then it can interpolate the data exactly and the previous strategy cannot be employed since the minimum of Eq. (65) is zero. A prototypical example (when trying to identify the ancestors of the variable x_1) is $K = K_s$=Eq. (47) with $\beta_3 > 0$. In this situation, we do not attempt to estimate the level of noise σ but select a prior γ such that the resulting noise-to-signal ratio can effectively differentiate noise from signal. To describe this, observe that the noise-to-signal ratio Eq. (62) admits the representer formula

$$\frac{\mathcal{V}(n)}{\mathcal{V}(s)+\mathcal{V}(n)} = \frac{Y^T D_\gamma^2 Y}{Y^T D_\gamma Y} \,, \tag{67}$$

involving the $N \times N$ matrix

$$D_\gamma := \gamma\big(K(X,X)+\gamma I\big)^{-1} \,. \tag{68}$$

Observe that $0 \le D_\gamma \le I$, and

$$\lim_{\gamma\downarrow 0} D_\gamma = 0 \text{ and } \lim_{\gamma\uparrow\infty} D_\gamma = I \,. \tag{69}$$

Write (λ_i, e_i) for the eigenpairs of $K(X, X)$ ($K(X, X)e_i = \lambda_i e_i$) where the λ_i are ordered in decreasing order. Then the eigenpairs of D_γ are (ω_i, e_i) where

$$\omega_i := \frac{\gamma}{\gamma + \lambda_i} \,. \tag{70}$$

Note that the ω_i are contained in $[0, 1]$ and also ordered in decreasing order.

Writing $\bar{Y}_i$ for the orthogonal projection of Y onto e_i, we have

$$\frac{\mathcal{V}(n)}{\mathcal{V}(s)+\mathcal{V}(n)} = \frac{\sum_{i=1}^{n} \omega_i^2 \bar{Y}_i^2}{\sum_{i=1}^{n} \omega_i \bar{Y}_i^2}\,, \qquad [71]$$

It follows that if the histogram of the eigenvalues of D_γ is concentrated near 0 or near 1, then the noise-to-signal ratio is non-informative since the prior γ dominates it. To avoid this phenomenon, we select γ so that the eigenvalues of D_γ are well spread out in the sense that the histogram of its eigenvalues has maximum or near-maximum variance (see Fig. S1 for a good choice and a bad choice for γ). If the eigenvalues have an algebraic decay, then this is equivalent to taking γ to be the geometric mean of those eigenvalues.
In practice, we use an off-the-shelf optimizer to obtain γ by maximizing the sample variance of $(\omega_i)_{i=1}^n$. If this optimization fails, we default to the median of the eigenvalues. This ensures a balanced, well-spread spectrum for $D\gamma$, with half of the eigenvalues λ_i being lower and half being higher than the median.

B.3. Rationale for the choices of γ**.** The purpose of this section is to present a rationale for the proposed choices for γ in Sec. B.1 and B.2. For the choice Sec. B.1, we present an asymptotic analysis of the signal-to-noise ratio in the setting of a simple linear regression problem. According to Eq. (66), γ must scale linearly in N; this scaling is necessary to achieve a ratio that represents the signal-to-noise per sample. Without it (if γ remains bounded as a function of N), this scaling of the signal-to-noise would converge towards 0 as $N \to \infty$. To see how we will now consider a simple example in which we seek to linearly regress the variable y as a function of the variable x, both taken to be scalar (in which case $\psi(x) = x$). Assume that the samples are of the form $Y_i = aX_i + \sigma Z_i$ for $i = 1, \ldots, N$, where $a, \sigma \neq 0$, the Z_i are i.i.d. $\mathcal{N}(0,1)$ random variables, and the X_i satisfy $\frac{1}{N}\sum_{i=1}^N X_i = 0$ and $\frac{1}{N}\sum_{i=1}^N X_i^2 = 1$. Then, the signal-to-noise ratio is $\frac{\mathcal{V}(s)}{\mathcal{V}(s)+\mathcal{V}(n)}$ with $\mathcal{V}(s) = |v|^2$ and $\mathcal{V}(n) = \frac{1}{\gamma}\sum_{i=1}^N |vX_i - Y_i|^2$ and v is a minimizer of

$$\min_{v\in\mathbb{R}} |v|^2 + \frac{1}{\gamma}\sum_{i=1}^{N} |vX_i - Y_i|^2\,. \qquad [72]$$

In asymptotic $N \to \infty$ regime, we have $v \approx \frac{aN}{\gamma+N}$ and

$$\frac{\mathcal{V}(s)}{\mathcal{V}(s)+\mathcal{V}(n)} \approx \frac{\frac{\gamma}{N}a^2}{-a^2(\gamma/N+1) + (a^2+\sigma^2)(\gamma/N+1)^2}\,. \qquad [73]$$

If γ is bounded independently from N, then $\frac{\mathcal{V}(s)}{\mathcal{V}(s)+\mathcal{V}(n)}$ converges towards zero as $N \to \infty$, which is undesirable as it does not represent a signal-to-noise ratio per sample. If $\gamma = N$, then $\frac{\mathcal{V}(s)}{\mathcal{V}(s)+\mathcal{V}(n)} \approx \frac{a^2}{4\sigma^2+2a^2}$, which does not converge to 1 as $a \to \infty$ and $\sigma \to 0$, which is also undesirable. If γ is taken as in Eq. (66), then $\gamma \approx N\sigma^2$ and

$$\frac{\mathcal{V}(s)}{\mathcal{V}(s)+\mathcal{V}(n)} \approx \frac{a^2}{(\sigma^2+1)(a^2+\sigma^2+1)}\,, \qquad [74]$$

which converges towards 0 as $\sigma \to \infty$ and towards $1/(1+\sigma^2)$ as $a \to \infty$, which has, therefore, the desired properties.

Moving to Sec. B.2, because the kernel can interpolate the data exactly we can no longer use Eq. (65) to estimate the level of noise σ. For a finite-dimensional feature map ψ, with data (X, Y), we can decompose $Y = v^T\psi(X) + \sigma Z$ into a signal part Y_s and noise part Y_s, s.t. $Y = Y_s + Y_n$. While Y_s belongs to the linear span of eigenvectors of $K(X, X)$ associated with non-zero eigenvalues, Y_n also activates the eigenvectors associated with with the null space of $K(X, X)$ and the projection of Y onto that null-space is what allows us to derive γ in Sec. B.1. Since in the interpolatory case, all eigenvalues are strictly positive, we need to choose which eigenvalues are associated with noise differently, as is described in the previous section. With a fixed γ, we see that if $\lambda_i \gg \gamma$, then $\omega_i \approx 0$, which contributes in (71) to yield a low noise-to-signal ratio. Similarly, if $\lambda_i \ll \gamma$, this eigenvalue yields a high noise-to-signal ratio. Thus, we see that the choice of γ assigns a noise level to each eigenvalue. While in the finite-dimensional feature map setting, this assignment is binary, here we perform soft thresholding using $\lambda \mapsto \gamma/(\gamma+\lambda)$ to indicate the level of noise of each eigenvalue. This interpretation sheds light on the selection of γ in equation Eq. (66). Let ψ represent the feature map associated with K. Assuming the empirical mean of $\psi(X_i)$ is zero, the matrix $K(X, X)$ corresponds to an unnormalized kernel covariance matrix $\psi^T(X)\psi(X)$. Consequently, its eigenvalues correspond to N times the variances of the $\psi(X_i)$ across various eigenspaces. After conducting Ordinary Least Squares regression in the feature space, if the noise variance is estimated as $\bar{\sigma}^2$, then any eigenspace of the normalized covariance matrix whose eigenvalue is lower than $\bar{\sigma}^2$ cannot be recovered due to the noise. Given this, we set the soft thresholding cutoff to be $\gamma = N\bar{\sigma}^2$ for the unnormalized covariance matrix $K(X, X)$.

C. Z-score/quantile bounds on the noise-to-signal ratio.. If the data is only comprised of noise, then an interval of confidence can be obtained on the noise-to-signal ratio. To describe this consider the problem of testing the null hypothesis $\mathbf{H_0} : f^\dagger \equiv 0$ (there is no signal) against the alternative hypothesis $\mathbf{H_1} : f^\dagger \not\equiv 0$ (there is a signal). Under the null hypothesis $\mathbf{H_0}$, the distribution of the noise-to-signal ratio Eq. (67) is known and it follows that of the random variable

$$B := \frac{Z^T D_\gamma^2 Z}{Z^T D_\gamma Z}\,. \qquad [75]$$

 Théo Bourdais, Pau Batlle, Xianjin Yang, Ricardo Baptista, Nicolas Rouquette, Houman Owhadi

Therefore, the quantiles of B can be used as an interval of confidence on the noise-to-signal ratio if $\mathbf{H_0}$ is true. More precisely, selecting β such that $\P[B \le \beta_\alpha] \approx \alpha$ with $\alpha = 0.05$ as a prototypical example, we expect the noise to signal ratio Eq. (67) to be, under $\mathbf{H_0}$, to be larger than β_α with probability $\approx 1-\alpha$. The estimation of β requires Monte-Carlo sampling.

An alternative approach (in the large data regime) to using the quantile β_α is to use the Z-score

$$\mathcal{Z} := \frac{\frac{Y^T D_\gamma^2 Y}{Y^T D_\gamma Y} - \mathbb{E}[B]}{\sqrt{\operatorname{Var}[B]}}\,, \qquad [76]$$

after estimating $\mathbb{E}[B]$ and $\operatorname{Var}[B]$ via Monte-Carlo sampling. In particular if $\mathbf{H_0}$ is true then $|\mathcal{Z}| \ge z_\alpha$ should occur with probability $\approx \alpha$ with $z_{0.1} = 1.65$, $z_{0.05} = 1.96$ and $z_{0.01} = 2.58$.

Remark 2. *Although the quantile β_α or the Z-score $\mathcal{Z}$ can be employed to produce an interval of confidence on the noise-to-signal ratio under $\mathbf{H_0}$ we cannot use them as thresholds for removing nodes from the list of ancestors as discussed in Sec. C.4 Indeed, observing a noise-to-signal ratio* Eq. (67) *below the threshold β_α does not imply that all the signal has been captured by the kernel; it only implies that some signal has been captured by the kernel K. To illustrate this point, consider the setting where one tries to approximate the variable x_1 as a function of the variable x_2. If x_1 is not a function of x_2, but of x_2 and x_3, as in $x_1 = \cos(x_2) + \sin(x_3)$, then applying the proposed approach with Y encoding the values of x_1, X encoding the values of x_2, and the kernel K depending on x_2 could lead to a noise-to-signal ratio below β_α due to the presence of a signal in x_2. Therefore, although we are missing the variable x_3 in the kernel K, we would still observe a possibly low noise-to-signal ratio due to the presence of* some *signal in the data. Summarizing if the data only contains noise then $\frac{\mathcal{V}(n)}{\mathcal{V}(s)+\mathcal{V}(n)} \ge \beta_\alpha$ should occur with probability $1-\alpha$. If the event $\frac{\mathcal{V}(n)}{\mathcal{V}(s)+\mathcal{V}(n)} < \beta_\alpha$ is observed in the setting of $K = K_{s/t}$=*Eq. (57) *where we try to identify the ancestors of x_1, then we can only deduce that $x_3, \ldots, x_d$ contain some signal but perhaps not all of it (we can use this a criterion for pruning x_2).*

8. Supplementary information on examples.

A. Algebraic equations.. Although we have used Alg. 2 for the algebraic equations examples presented in Fig. 4, Alg. 1 yields the same results with the default signal-to-noise threshold $\tau = 0.5$.

B. The chemical reaction network.. Consider the chemical reaction network example illustrated in Fig. 4.(a). The proposed mechanism for the hydrogenation of ethylene (C_2H_4) to ethane (C_2H_6), is (writing $[H]$ for the concentration of H) modeled by the following system of differential equations

$$\begin{aligned}
\frac{d[H_2]}{dt} &= -k_1[H_2] + k_{-1}[H]^2\\
\frac{d[H]}{dt} &= 2k_1[H_2] - 2k_{-1}[H]^2 - k_2[C_2H_4][H] - k_3[C_2H_5][H]\\
\frac{d[C_2H_4]}{dt} &= -k_2[C_2H_4][H]\\
\frac{d[C_2H_5]}{dt} &= k_2[C_2H_4][H] - k_3[C_2H_5][H]
\end{aligned} \qquad [77]$$

The primary variables are the concentrations $[H_2]$, $[H]$, $[C_2H_4]$ and $[C_2H_5]$ and their time derivatives $\frac{d[H_2]}{dt}$, $\frac{d[H]}{dt}$, $\frac{d[C_2H_4]}{dt}$ and $\frac{d[C_2H_5]}{dt}$. The computational hypergraph encodes the functional dependencies Eq. (77) associated with the chemical reactions. The hyperedges of the hypergraph are assumed to be unknown and the primary variables are assumed to be known. Given N samples from the graph of the form

$$\big([H_2](t_i), [H](t_i), [C_2H_4](t_i), [C_2H_5](t_i)\big)_{i=1,\ldots,N} \qquad [78]$$

our objective is to recover the structure of the hypergraph given by Eq. (77), representing the functions by hyperedges. We create a dataset of the form Eq. (78) by integrating 50 trajectories of Eq. (77) for different initial conditions, and each equispaced 50 times from $t = 0$ to $t = 5$. The dataset is represented in Fig. 4.(b) (the time derivatives of concentrations are estimated by taking the derivatives of the interpolants of those concentrations). We impose the information that the derivative variables are function of the non-derivative variables to avoid ambiguity in the recovery, as Eq. (77) is not the unique representation of the functional relation between nodes in the graph. We implement Alg. 1 with weights $\beta = [0.1, 0.01, 0.001]$ for linear, quadratic, and nonlinear, respectively (Alg. 2 recovers the same hypergraph). The output graph can be seen in Fig. 4.(b). We obtain a perfect recovery of the computational graph and a correct identification of the relations being quadratic.

C. The Google Covid 19 open data.. Consider the example illustrated in Fig. 3.(e-k). Categorical data are treated as scalar values, with all variables scaled to achieve a mean of 0 and a variance of 1. We implement three distinct kernel types: linear, quadratic, and Gaussian, with a length scale of 1 for the latter. A weight ratio of 1/10 is assigned between kernels, signifying that the quadratic kernel is weighted ten times less than the linear kernel. Lastly, the noise parameter, γ, is determined using the optimal value outlined in Sec. 7. Initially, a complete graph is constructed using all variables, depicted in Fig. 3.(g). This

construction is done using only linear and quadratic kernels. The full graph is highly clustered and redundant information is eliminated by selecting representative nodes for each cluster. Eliminating redundant nodes is important for two reasons: firstly, it improves the graph's readability, especially with 31 variables; secondly, it avoids hindering graph discovery. In an extreme case, treating two identical variables as distinct would result in one variable's ancestor simply being its duplicate, yielding an uninformative graph. Subsequently, the graph discovery algorithm is rerun, with reduced variables due to eliminating redundancy, ushering us into a predominantly noisy regime. With fewer variables available, we use additionally the nonlinear kernel. Two indicators are employed to navigate our discovery process: the signal-to-noise ratio and the Z-test. The former quantifies the degree to which our regression is influenced by noise, while the latter signals the existence of any signal. We follow the procedure in algorithm 2, resulting in the graph presented in Fig. 3.(k).

D. Cell signaling network. Consider the example Fig. 1.(l) from (22) and Fig. 4.(h-j). To identify the ancestors of each node, we apply the algorithm in two stages. First, we learn the dependencies using only linear and quadratic kernels. Fig. 4.(h) identifies the resulting graph learned given a subset of $N = 2,000$ samples chosen uniformly at random from the dataset. We observe that the graph identified by the algorithm consists of four disconnected clusters where the molecule levels in each cluster are closely related by linear or quadratic dependencies (all connections are linear except for the connection between Akt and PKA, which is quadratic). These edges match a subset of the edges found in the gold standard model identified in (22). With perfect dependencies that have no noise, one can define constraints that reduce the total number of variables in the system. For this noisy dataset that, we treat these dependencies as forming groups of similar variables and introduce a hierarchical approach to learn the connections between groups. Second, we run the graph discovery algorithm after grouping the molecules into clusters. For each node in the graph, we identified the ancestors of each node by constraining the dependence to be a subset of the clusters. In other words, when identifying the ancestors of a given node i in cluster C, the algorithm is only permitted to (1) use ancestors that do not belong to cluster C, and (2) include all or none of the variables in each cluster (j in cluster $D \neq C$ is listed as an ancestor if and only if all other nodes j' in cluster D are also listed as ancestors). The ancestors were identified using a Gaussian (fully nonlinear) kernel and the number of by ancestors were selected manually based on the inflection point in the noise-to-signal ratio. The resulting graph is depicted in Fig. 4.(i). Each edge is weighted based on its signal-to-noise ratio. We observe that there is a stronger dependence of the Jnk, PKC, and P38 cluster on the PIP3, Plcg, and PIP2 cluster, which closely matches the gold standard model. As compared to approaches based on acyclic DAGs, however, the graph identified by our algorithm also contains feedback loops between the various molecule levels. Fig. 4.(i-j) displays a side-by-side comparison between the graph identified with our method and the graph generated in (22). To aid in this comparison, we have highlighted different clusters in distinct colors. We emphasize that while the Bayesian network analysis in (22) relied on the control of the sampling of the underlying variables (the simultaneous measurement of multiple phosphorylated protein and phospholipid components in thousands of individual primary human immune system cells, and perturbing these cells with molecular interventions), the reconstruction obtained by our method did not use this information and recovered functional dependencies rather than causal dependencies. Interestingly, the information recovered through our method appears to complement and enhance the findings presented in (22) (e.g., the linear and noiseless dependencies between variables in the JNK cluster is not something that could easily be inferred from the graph produced in (22)).

E. BCR reaction network. In the high-dimensional example of the BCR reaction network, the computations of terms of the form $y^T k_o(X,X) y$ (i.e., the activations), where $y \in \mathbb{R}^n$ and $k_o(X,X)$ is the o-th coordinate of the quadratic kernel ($k(x_i,x_j) = (1+\langle x_i, x_j\rangle)^2$) becomes the computational bottleneck of our method. If we let $x_1,\dots,x_n \in \mathbb{R}^p$ be the points and x_i^o be the o-th coordinate of x_i, we can compute the activation of the $o-$th coordinate using

$$k_o(x_i,x_j) = (1 + x_i^o x_j^o)^2 - 1 + 2x_i^o x_j^o \langle x_i^{-o}, x_j^{-o}\rangle \tag{79}$$

where k_o is the $o-$th coordinate of the kernel and x_i^{-o} represents the remaining coordinates of x_i. To compute the $n \times n$ kernel matrix of k_o for each $o \in \{1,..,p\}$, we must compute $p \times n \times n$ inner products in $\mathbb{R}^p$, which is a very large computation. Instead, we may use the following reformulation to speed up computations. Notice $\langle x_i, x_j\rangle = x_i^o x_j^o + \langle x_i^{-o}, x_j^{-o}\rangle$, and therefore $k_o(x_i,x_j) = 2x_i^o x_j^o \langle x_i, x_j\rangle + 2x_i^o x_j^o - (x_i^o x_j^o)^2$. Now, define $v^o = (x_i^o y_i)_{i=1}^p$ and $w^o = ((x_i^o)^2 y_i)_{i=1}^p$, and note that

$$y^T K_o y = \sum_{i,j} 2y_i x_i^o y_j x_j^o (1 + \langle x_i, x_j\rangle) - \sum_{i,j} y_i y_j (x_i^o x_j^o)^2 \tag{80}$$

and so defining $\tilde{K} = (2(1+\langle x_i, x_j\rangle))_{i,j=1}^n$ we have that

$$y^T K_o y = v^{oT} \tilde{K} v^o - \left(\sum_{i=1}^p w_i^o\right)^2 \tag{81}$$

Note that $\tilde{K}$ is computed just once for all p, and only v^o and w^o change for every ancestor calculation, which is where the main computational gain comes from. One may find in the GitHub repository of the paper a comparison of the two methods of computations and observe a tenfold speedup. This speedup is even larger in our implementation of the BCR example, as GPU acceleration enables the second method to run even faster.

References

1. S Mika, et al., Kernel pca and de-noising in feature spaces. in *NIPS*. Vol. 11, pp. 536–542 (1998).
2. H Owhadi, C Scovel, GR Yoo, *Kernel Mode Decomposition and the programming of kernels.* (Springer), (2021).
3. H Owhadi, Computational graph completion. *Res. Math. Sci.* **9**, 1–33 (2022).
4. T Ishihara, Enumeration of hypergraphs. *Eur. J. Comb.* **22**, 503–509 (2001).
5. P Spirtes, C Glymour, An algorithm for fast recovery of sparse causal graphs. *Soc. science computer review* **9**, 62–72 (1991).
6. DM Chickering, Optimal structure identification with greedy search. *J. machine learning research* **3**, 507–554 (2002).
7. J Peters, D Janzing, B Schölkopf, *Elements of causal inference: foundations and learning algorithms.* (The MIT Press), (2017).
8. MC Data, JD Salciccioli, Y Crutain, M Komorowski, DC Marshall, Sensitivity analysis and model validation. *Second. analysis electronic health records* pp. 263–271 (2016).
9. M Drton, MH Maathuis, Structure learning in graphical modeling. *Annu. Rev. Stat. Its Appl.* **4**, 365–393 (2017).
10. J Friedman, T Hastie, R Tibshirani, Sparse inverse covariance estimation with the graphical lasso. *Biostatistics* **9**, 432–441 (2008).
11. R Baptista, Y Marzouk, RE Morrison, O Zahm, Learning non-gaussian graphical models via hessian scores and triangular transport. *arXiv preprint arXiv:2101.03093* (2021).
12. CX Ren, S Misra, M Vuffray, AY Lokhov, Learning continuous exponential families beyond gaussian. *arXiv preprint arXiv:2102.09198* (2021).
13. K Zhang, J Peters, D Janzing, B Schölkopf, Kernel-based conditional independence test and application in causal discovery in *27th Conference on Uncertainty in Artificial Intelligence (UAI 2011).* (AUAI Press), pp. 804–813 (2011).
14. RD SHAH, J PETERS, The hardness of conditional independence testing and the generalised covariance measure. *The Annals Stat.* **48**, 1514–1538 (2020).
15. F Schäfer, TJ Sullivan, H Owhadi, Compression, inversion, and approximate pca of dense kernel matrices at near-linear computational complexity. *Multiscale Model. & Simul.* **19**, 688–730 (2021).
16. F Schäfer, M Katzfuss, H Owhadi, Sparse cholesky factorization by kullback–leibler minimization. *SIAM J. on Sci. Comput.* **43**, A2019–A2046 (2021).
17. AV Vecchia, Estimation and model identification for continuous spatial processes. *J. Royal Stat. Soc. Ser. B: Stat. Methodol.* **50**, 297–312 (1988).
18. G Wahba, An introduction to smoothing spline anova models in rkhs, with examples in geographical data, medicine, atmospheric sciences and machine learning. *IFAC Proc. Vol.* **36**, 531–536 (2003).
19. IM Sobol, Global sensitivity indices for nonlinear mathematical models and their monte carlo estimates. *Math. computers simulation* **55**, 271–280 (2001).
20. I Sobol̆, Sensitivity estimates for nonlinear mathematical models. *Math. Model. Comput. Exp.* **1**, 407 (1993).
21. AB Owen, Variance components and generalized sobol'indices. *SIAM/ASA J. on Uncertain. Quantification* **1**, 19–41 (2013).
22. K Sachs, O Perez, D Pe'er, DA Lauffenburger, GP Nolan, Causal protein-signaling networks derived from multiparameter single-cell data. *Science* **308**, 523–529 (2005).
